# Comparative Proteomic Analysis of Lung Lamellar Bodies and Lysosome-Related Organelles

**DOI:** 10.1371/journal.pone.0016482

**Published:** 2011-01-26

**Authors:** Ross Ridsdale, Cheng-Lun Na, Yan Xu, Kenneth D. Greis, Timothy Weaver

**Affiliations:** 1 Division of Pulmonary Biology, Cincinnati Children's Hospital Medical Center, and Department of Pediatrics, University of Cincinnati College of Medicine, Cincinnati, Ohio, United States of America; 2 Department of Cancer and Cell Biology, University of Cincinnati College of Medicine, Cincinnati, Ohio, United States of America; McMaster University, Canada

## Abstract

Pulmonary surfactant is a complex mixture of lipids and proteins that is essential for postnatal function. Surfactant is synthesized in alveolar type II cells and stored as multi-bilayer membranes in a specialized secretory lysosome-related organelle (LRO), known as the lamellar body (LB), prior to secretion into the alveolar airspaces. Few LB proteins have been identified and the mechanisms regulating formation and trafficking of this organelle are poorly understood. Lamellar bodies were isolated from rat lungs, separated into limiting membrane and core populations, fractionated by SDS-PAGE and proteins identified by nanoLC-tandem mass spectrometry. In total 562 proteins were identified, significantly extending a previous study that identified 44 proteins in rat lung LB. The lung LB proteome reflects the dynamic interaction of this organelle with the biosynthetic, secretory and endocytic pathways of the type II epithelial cell. Comparison with other LRO proteomes indicated that 60% of LB proteins were detected in one or more of 8 other proteomes, confirming classification of the LB as a LRO. Remarkably the LB shared 37.8% of its proteins with the melanosome but only 9.9% with lamellar bodies from the skin. Of the 229 proteins not detected in other LRO proteomes, a subset of 34 proteins was enriched in lung relative to other tissues. Proteins with lipid-related functions comprised a significant proportion of the LB unique subset, consistent with the major function of this organelle in the organization, storage and secretion of surfactant lipid. The lung LB proteome will facilitate identification of molecular pathways involved in LB biogenesis, surfactant homeostasis and disease pathogenesis.

## Introduction

The lung is composed of a tubular branching network that terminates in sac-like structures (alveoli) where gas-exchange occurs. The air-blood barrier consists of thin type I epithelial cells that cover 95% of the alveolar surface [1], a basal lamina and the underlying capillary endothelium. Alveolar structure is critically dependent on the spreading of a phospholipid-rich film, pulmonary surfactant, at the air/liquid interface on the epithelial side of the air-blood barrier [2], [3]. Surfactant insufficiency, due to premature birth, or inactivation, due to acute lung injury, results in increased surface tension and alveolar collapse leading to decreased gas exchange and respiratory distress syndrome [4].

Surfactant is a mixture of proteins (1–2% surfactant protein (SP)-B and SP-C) and lipids (80% phospholipids and ∼10% neutral lipids) that is synthesized by alveolar type II epithelial cells [5]. The complex is stored in specialized secretory organelles (lamellar bodies, LB) in the form of tightly packed, concentric bilayer membranes. Regulated release of surfactant into the airspaces maintains the alveolar surfactant film, which is continually turned over [6]. Spent alveolar surfactant is degraded by alveolar macrophages or internalized by type II cells for degradation or recycling back to the LB [7]. Therefore, the LB plays an important role in integrating the surfactant biosynthetic and recycling pathways to maintain the intracellular surfactant pool.

The process of LB maturation is poorly understood [5]. Surfactant phospholipids are synthesized in the ER but the pathway(s) by which they are transported to and selectively accumulated in LBs is not clear. ABCA3 likely plays an important role transporting phosphatidylcholine (PC) and phosphatidylglycerol (PG) across the limiting membrane and into the LB [8]–[12]: however, it is not known if other lipid “importers” reside in the LB membrane or if there is a retrograde transport pathway for removal of specific lipid species from the LB. Also, the mechanism of surfactant lipid incorporation into highly organized membranes is not well understood, although SP-B certainly contributes to this process [13], [14]. Importantly it is not known how the biosynthetic and recycling pathways are integrated to maintain cellular surfactant homeostasis. As a first step towards answering these and other questions related to LB biogenesis, we have identified the rat LB proteome. In all, 562 LB proteins were identified, significantly expanding a previous study that detected 44 proteins [15].

## Materials and Methods

Antibodies directed against mature SP-B, SP-C, ABCA3, lysozyme and β actin were obtained from Seven Hills Bioreagents (Cincinnati, Ohio). Anti-calnexin and anti-PDIA3 antibodies were obtained from Sigma-Aldrich (St. Louis, MO). Anti-BiP, anti-EHD2 and anti-EHD4 antibodies were obtained from Stressgen Bioreagents (Ann Arbor, MI), Imgenex (San Diego, Ca), and Novus Biologicals (Littleton, CO) respectively. Anti-AGER antibody was purchased from Abcam Inc. (Cambridge, MA).

### Lamellar body isolation

Lamellar bodies were isolated from rat lungs as described by Gil and Reiser [16] and modified by Jobe, *et al.* [17]. Adult male Sprague Dawley rats (200–300 grams) were purchased from Charles River. Rats were euthanized and the lungs were repeatedly lavaged with 5 ml of PBS via an endotracheal catheter to remove extracellular surfactant. The first two bronchoalveolar lavage fluid (BALF) samples were centrifuged at 500×*g* for ten minutes and saved for western blot analysis. Lung tissue was minced and homogenized in 250 mM sucrose/PBS and centrifuged in a swinging bucket rotor for 5 minutes at 1000×*g* at 4°C. The resultant supernatant was layered on top of a discontinuous PBS/sucrose gradient and centrifuged at 100,000×*g* for 60 minutes at 4°C in a Beckmann Optima L centrifuge with a SW28 swinging bucket. The lamellar body fraction was recovered at the interface between 0.45 M and 0.65 M sucrose. All animal protocols were approved by The Institutional Animal Care and Use Committee (protocol #0D04031) and complied with the American Association of Laboratory Animal Care guidelines and the relevant NIH regulations.

### Limiting membrane protein isolation

Isolated LBs were incubated for 30 minutes with 10 mM sulfo-NHS-SS-Biotin in saline on ice. Excess glycine was used to consume unreacted sulfo-NHS-SS-Biotin. The samples were treated with an equal volume of 0.2 M Na_2_CO_3_ (pH 11.3) in saline for 30 minutes on ice to remove peripheral membrane proteins. Stripped lamellar bodies were centrifuged at 100,000×*g* for 60 minutes, 4°C using a SW55TI swinging bucket. The pellet was washed with PBS, and then lysed with RIPA buffer. To isolate biotinylated proteins, the lysate was passed through a tetralink tetrameric avidin resin (Promega, Madison, WI) column. Protein that did not bind to the column was kept for analysis of the inner core of the LB. Limiting membrane proteins were eluted from the column using β-mercaptoethanol in 25 mM ammonium bicarbonate.

### Protein separation, digestion and identification

In order to maximize the number of proteins that could be identified from each of the LB subfractions, multiple sample preparation strategies were employed prior to mass spectrometry. For the primary approach, LB, limiting membrane enriched (LME), and limiting membrane depleted (LMD) fractions were solubilized directly in Laemmli buffer, separated by SDS-PAGE on a 16 by 18 cm 4–20% Tris/Glycine gel (Jules Biotechnologies Inc., Milford, CT), run on a Hoefer SE400 electrophoresis system (Hoefer Inc., Holliston, MA) and visualized by Imperial protein stain (Thermo Fisher Scientific Inc., Rockford, IL). Each lane was then cut into a grid of 15, 0.5 cm segments. The 15 samples from each lane were prepared for mass spectrometry by reduction with dithiothreitol, alkylation with iodoacetamide, trypsin digestion and peptide recovery, all as described previously [18]. The resulting peptides from each gel region were concentrated in a SpeedVac and analyzed by nanoLC-MSMS on a ThermoFisher LTQ linear ion trap mass spectrometer equipped with an Eksigent nanoLC system and a 150×0.1 mm C18 Chromolith® monolithic column (Merck KGaA, Darmstadt, Germany). The LTQ MS system was optimized such that peptide signals that produce ion intensities at 1.5 times above the noise level were subjected to automated MS/MS fragmentation. Individual ion masses were permitted to be captured and fragment twice after which they were subsequently excluded for 1.5 minutes. This method produced >10,000 fragmentations with sub-femtomole sensitivity over a typical 90 minute nanoLC-MS/MS separation. The collective MS/MS spectral data from the 15 gel regions for each of the LB fractions were merged and searched against NCBInr database (filtered for rodent entries) for protein identification using the Mascot (Matrix Science, Boston, MA) search algorithm on an in house 8 processor proteomics server. Positive protein identifications were based on a total Mascot score ≥75 with a p-value at 0.005.

Given the high lipid content of LBs and the number of known hydrophobic proteins present in these fractions, intact LBs were also subjected to pressure cycling technology (PCT) extraction using a Barocycler NEP2320 (Pressure BioSciences Inc., (PBI), South Easton, MA) and the ProteoSolve-SB reagent kit (PBI). Briefly, isolated LBs (∼1 mg protein) were concentrated into PBI Pulse tubes by SpeedVac, resuspended in ProteoSolve-SB, subjected to 20 cycles of 35,000 psi pulse for 20 seconds each to disrupt the tissue and the resulting protein extracts isolated by centrifugation [19]. The protein fraction was dried, dissolved in Laemmli buffer and separated by SDS-PAGE as described above. Gridded sections of the gel were prepared, digested and identified by mass spectrometry as described above.

### Transmission Electron Microscopy (TEM)

Tissue, LB processing and immunogold labeling were performed as previously described [20]. Electron micrographs of LBs were acquired at a magnification of 80,000X using a Hitachi H-7600 TEM (Hitachi High- Technologies America, Inc., Pleasanton, CA) equipped with a 2000 by 2000 pixel TEM CCD digital camera (Advanced Microscopy Techniques, Danvers, MA). To determine if BiP, mature SP-B, or ABCA3 localized to specific compartments in the LB, gold counts registered on the limiting membrane and lamellar lipid layer were tabulated and analyzed by the relative labeling index (RLI), as described by Mayhew *et al*. [21]. LB compartments that had higher than expected RLI (p-value<0.05) and significantly higher partial χ^2^ values compared to other compartments were considered to be significantly labeled.

### Real Time Polymerase Chain Reaction

Tissues were collected from embryonic (E) mice, flash frozen in liquid nitrogen and stored at −80°C. Type II cells were isolated from 6 week old female mice as previously described [22]. Messenger RNA was isolated from cells/tissue using RNeasy (Qiagen, Valencia, CA). Complementary DNA was generated using iScript (BioRad, Hercules, CA). Real time polymerase chain reaction was performed with 25 ng/sample cDNA on a 7300 Real-Time PCR System using Taqman gene expression primers (Applied Biosystems Inc. Foster City, CA). Relative quantification of transcripts was determined by normalization to β-actin.

## Results

Proteomic analyses were performed on lamellar bodies isolated from rat lungs. Of particular interest to these analyses were the proteins of the limiting membrane, as this subpopulation is likely involved in the biogenesis and maturation of the organelle. To enrich for limiting membrane proteins, surface accessible free amines on isolated LBs were conjugated to sulfo-NHS-SS-biotin. Biotinylated LBs were subsequently washed with Na_2_CO_3_ to strip cytoskeletal proteins, lysed in RIPA buffer and loaded onto an avidin column. The column was washed extensively and bound proteins were eluted by reduction of the biotin conjugate. Proteins not captured by avidin represented enrichment of the interior, core LB proteins, while the eluted fraction represented enrichment of LB limiting membrane proteins (Figure S1). Lamellar bodies lysed before or during biotinylation resulted in some core proteins being labeled and co-isolated with the limiting membrane fraction.

**Figure 1:**
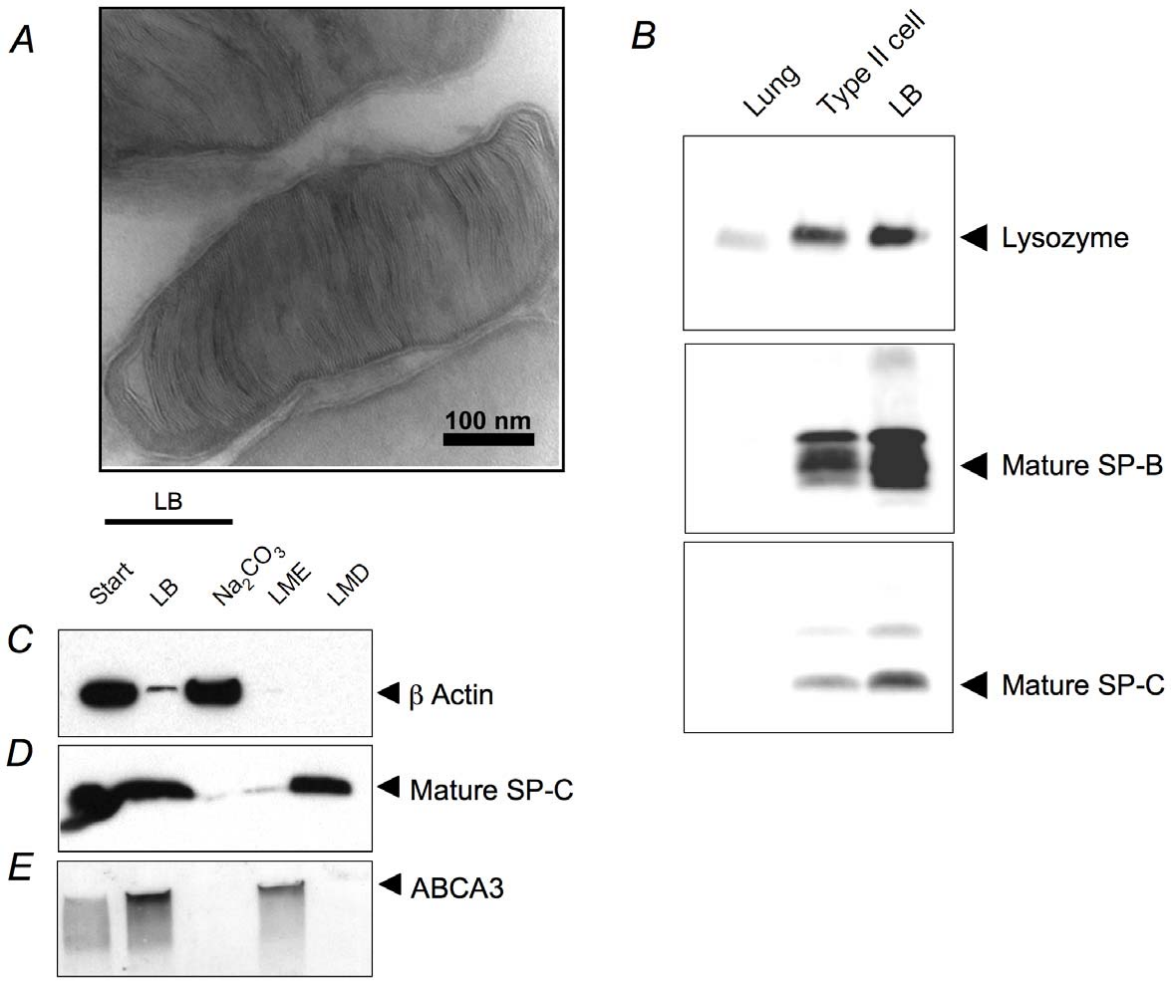
Characteristics of isolated lamellar bodies. (A) Cryo-TEM of LB samples obtained before Na_2_CO_3_ stripping or sulfo-NHS-SS-biotin treatment. (B) Enrichment of lysozyme, mature SP-B and mature SP-C during LB isolation. (C–E) The effect of Na_2_CO_3_ mediated peripheral protein stripping on LB protein distribution. **Start** is the initial LB sample, **LB** is the LB sample after Na_2_CO_3_ treatment, **Na_2_CO_3_** is the Na_2_CO_3_ solution after stripping, LME refers to limiting membrane enriched fraction and LMD refers to limiting membrane depleted fraction. (C) β-actin protein, (D) mature SP-C and (E) ABCA3 protein within these fractions. All samples were normalized to total protein content.

**Figure 2:**
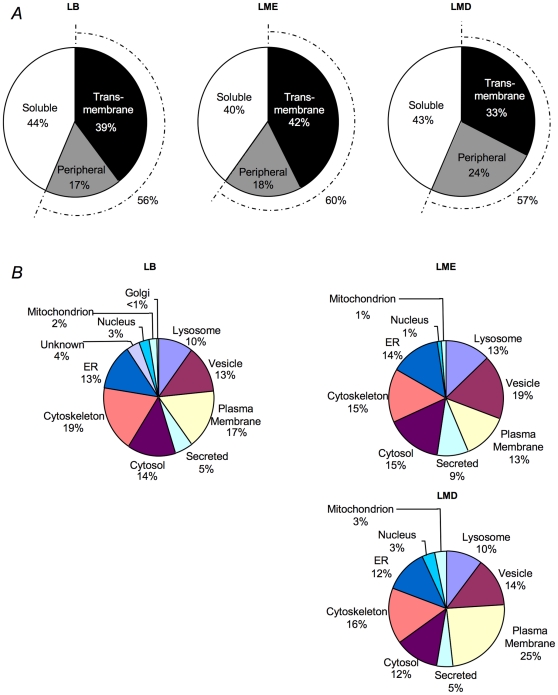
Characteristics of LB proteome. (A) Distribution of membrane proteins in the proteome with respect to the 3 LB fractions. (B) Predicted subcellular localization of proteins in the LB, LMD and LME fractions.

The purity and integrity of the LB preparations was assessed by western blotting and electron microscopy. Cryo-TEM performed on LBs prior to Na_2_CO_3_ stripping detected largely intact organelles with organized lamellae and continuous limiting membranes (Figure 1A). The lamellar body proteins, surfactant protein B (SP-B), lysozyme and surfactant protein C (SP-C) (Figure 2B) were progressively enriched in lung homogenates, type II cells and isolated LBs, consistent with significant purification of the LB isolate. Subsequent treatment of LBs with Na_2_CO_3_ further enhanced purity: sodium carbonate stripping greatly reduced the content of actin (Figure 1C) and Clic5 (not shown) in the LB and limiting membrane fractions with consequent enrichment in the stripping buffer. In contrast SP-C (Figure 1D) was not removed by stripping buffer and was detected in the limiting membrane depleted (LMD) fraction. The LB limiting membrane protein, ABCA3 (Figure 1E), was not removed by Na_2_CO_3_ stripping and was recovered in the limiting membrane enriched (LME) fraction, as expected. Together these results are consistent with minimal perturbation of LB structure during the isolation procedure.

The LB proteome was compiled from MS analyses of the three LB fractions (intact LB, LME and LMD). A total of 455 proteins were detected at least once with a Mascot score ≥75: 351 proteins were detected in the intact LB fraction and 280 proteins were detected in the LME and LMD fractions (184 and 96 proteins, respectively). Many proteins were identified in both the LMD and LME fractions, likely related to failure of some proteins to bind the avidin matrix or retention of biotinylated core proteins on the column, respectively. Proteins were therefore assigned as LB limiting membrane (157) or LB core (87 proteins) based on their Mascot scores (Table S1); 211 proteins were not assigned to either compartment because the protein was detected only in the intact LB fraction or because the LME and LMD Mascot scores were very similar. Ribosomal proteins (13 in total, Table S2) were excluded in subsequent tables and analyses because these proteins are contaminants of many organelle proteomes [23]. As an alternative method to identify LB proteins, PCT was performed on stripped LBs to delipidate the sample prior to SDS-PAGE and MS analysis. Ninety-four of the 291 proteins detected by this method (Table S3) were not detected by the conventional approach, bringing the total number of proteins to 562. The entire 562 protein dataset was then used to interrogate archived microarrays of isolated, mouse type II epithelial cells (n = 8; [24] and unpublished data, available upon request). Twenty-three proteins (4%) were not represented on the Affymetrix chip and thus expression data are unavailable for this subset, while another 65 proteins (12%) were not expressed or expressed at low levels in the type II cell. Expression of the remaining 474 proteins (88%) was confirmed in type II cells. The broad representation of type II cell proteins in the LB proteome further confirms successful isolation of LBs from lung homogenates.

Like other organelles, the limiting membrane of the LB is expected to have a substantial number of transmembrane proteins; however, because the LB is filled with tightly packed bilayer membranes, the core protein transmembrane content is also expected to be very high. Proteins in each fraction were categorized as soluble, membrane-associated or membrane using the gene ontology database (http://www.ebi.ac.uk/GOA/). Each fraction, including the core LMD fraction, contained 33 to 42% transmembrane proteins (Figure 2A). These ranges are consistent with the previously reported ranges for ER and lysosomal membrane proteins [23]. The content of hydrophobic proteins (transmembrane + membrane associated) was very similar for the LME (60%) and LMD (57%) fractions consistent with the membrane cargo of the LB.

In order to assess the potential contribution of other organelles to the LB proteome, the subcellular localization of each protein was identified using the Gene Ontology Annotation Database and by cross-referencing with proteomes of other lysosome-related organelles (Figure 2B and Table S2). Lamellar bodies are lysosomal-like organelles that communicate extensively with both the bioysynthetic and endocytic pathways. Lysosomal proteins were well-represented in all three fractions as were proteins associated with the secretory and endocytic pathways (plasma membrane, secreted, vesicle and ER). Only a small number of proteins (<6%) were identified from the nucleus, mitochondria or Golgi consistent with minimal contamination from these compartments. The high concentration of cytoskeletal proteins was expected as the LB surface is densely covered by actin (see Figure 1C) [25].

**Figure 3:**
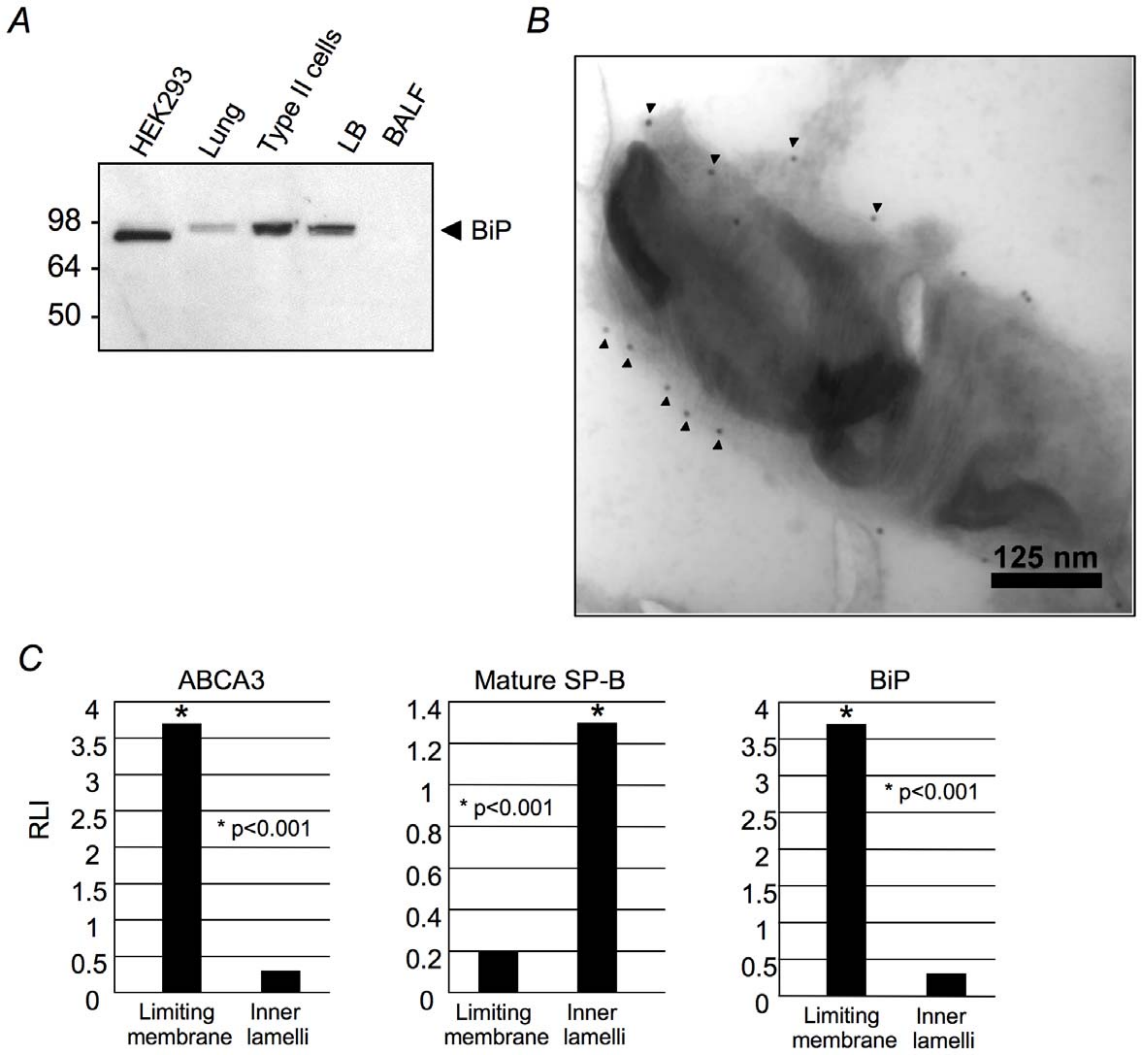
BiP distribution in LBs. (A) Western blot for BiP in HEK293 cells, whole lung, isolated type II cells, isolated LBs and BALF. Samples were normalized to protein content. (B) Immunogold labeling for BiP in isolated rat LB. (C) Relative labeling index (RLI) of isolated rat LBs labeled by immunogold for ABCA3, mature SP-B and BiP.

As with other lysosome-like organelles, a large number of ER proteins were detected in the LB proteome. Persistent co-purification of ER proteins could represent microsomal contamination and/or true localization in the LB. To begin to address this issue the distribution of the abundant ER chaperone BiP (Mascot score  = 1415) was assessed in intact lamellar bodies. BiP was detected by western blotting of lung homogenates, isolated type II cells and isolated LB but not BALF (Figure 3A). Immunogold labeling of isolated LBs (Figure 3B) and lung tissue sections (not shown) demonstrated that BiP was peripherally distributed in the LB in close approximation to the limiting membrane. Quantitative analyses of gold particles provided further support for this conclusion (Figure 3C). BiP counts were detected primarily at the limiting membrane (P<0.001) similar to ABCA3, a known LB limiting membrane protein: in contrast, SP-B counts were predominantly distributed over the internal membranes (P<0.001) as expected (Figure 3C). These findings, coupled, with lack of detection of BiP in BALF (Figure 3A), suggested that this ER protein accumulates in the LB but is not secreted, perhaps because of its association with the inner surface of the limiting membrane. In addition to BiP, calnexin (Mascot score  = 438) was also detected in LBs by immunogold labeling of isolated LBs (Figure S2A). Further, isoforms of the ER chaperone, protein disulfide iscmerase family A, member 3 (PDIA3, Mascot score  = 882), were detected in LBs by confocal microscopy of lung tissue sections (Figure S2B). Given these preliminary findings, the significant enrichment of ER proteins in the LB (Table S4) and the presence of ER proteins in the proteomes of 7 other LROs [23], we conclude that a subset of ER proteins is present in the LB. The number of ER proteins resident in the LB and the manner in which they are transported between the two organelles remains to be established.

**Figure 4:**
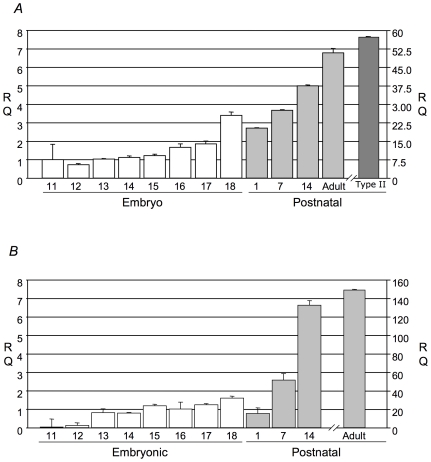
Relative mRNA expression levels of (A) ATP8A1 and (B) ABCA8a, as assessed by real-time polymerase chain reaction. Data is expressed as relative quantification (RQ), which is the fold difference in expression between embryonic day 11 sample with respect to all other samples.

Lamellar body proteins not present in other lysosome related organelles (LRO) proteomes are collected in Table 1. The major function of the LB is the assembly and storage of surfactant phospholipids for regulated release into the alveolar airspaces. Consistent with this function many of the proteins unique to the LB are involved in lipid or ion transport. In addition to ABCA3 (Mascot score  = 673) these include ATP8A1 (Mascot score  = 163), ABCA8a (Mascot score  = 107), StarD9 (Mascot score  = 76), Deleted in Liver Cancer 1/StarD12 (Mascot score  = 75), ATP1a1 (Mascot score  = 672), Major Vault Protein (Mascot score  = 546), SLC4A1 (Mascot score  = 393), and ATP2B4 (Mascot score  = 364). ATP8A1 and ABCA8a were the focus of further study due to their potential lipid transport activities. ABCA8a is a poorly characterized member of the ABCA subfamily of lipid transporters. MS analyses detected ABCA8a exclusively in the LB limiting membrane fraction (Table S1). Expression of ABCA8a mRNA in lung tissue increased modestly between E13-E17 with a more pronounced increased in the perinatal period (E18-PND1) and a significant increase in juvenile and adult lung (Figure 1A). Similarly, expression of mRNA encoding ATP8A1, increased from E16-E18 with a more pronounced increase in postnatal lung tissue (Figure 4A). ATP8A1 has been implicated in transport of several phospholipids species and, like ABCA8a, was exclusively detected in the LME fraction. Identification of these candidate phospholipid transport/transfer proteins is consistent with their predicted role in transport of lipid substrates across the LB limiting membrane.

**Table 1:** Proteins unique to the LB relative to other LROs.

			Mascot Score		
Rat Symbol	Human Symbol	NAME	LB	LM	Core
ABCA3	ABCA3	ATP-binding cassette, sub-family A (ABC1), member 3	272	673	120
ABCA8A	ABCA8A	ATP-binding cassette sub-family A member 8-A		107	
AGER	AGER	Advanced glycosylation end product-specific receptor	179	123	
ALCAM	ALCAM	Activated leukocyte cell adhesion molecule	161		
ATP8A1	ATP8A1	ATPase, aminophospholipid transporter (APLT), Class I, type 8A, member 1	163	119	
BCAM	BCAM	Basal cell adhesion molecule	181	148	
Car4	CAR4	Carbonic anhydrase 4 *	347		
CES3	CES3	Carboxylesterase 3	556		
CP	CP	Ceruloplasmin	905		136
CRIP2	CRIP2	Cysteine-rich protein 2		75	
CYP2F2	CYP2F2	Cytochrome P450, family 2, subfamily f, polypeptide 2	252		
CYP2S1	CYP2S1	Cytochrome P450, family 2, subfamily S, polypeptide 1	139		
CYP4B1	CYP4B1	Cytochrome P450, family 4, subfamily B, polypeptide 1	174	75	177
EHD2	EHD2	EH-domain containing 2	1371	447	384
ENPEP	ENPEP	Glutamyl aminopeptidase	500	261	
Fabp1	FABP1	Fatty acid binding protein 1, liver *	840		
FMO1	FMO1	Flavin containing monooxygenase 1	268		
Gstm2	GSTM2	Glutathione S-transferase, mu 2 *	276		
ITGA3	ITGA3	Integrin, alpha 3		94	
JUP	JUP	Junction plakoglobin	160		
LMO7	LMO7	LIM domain 7		75	
LYZ	LYZ	Lysozyme	199	98	232
MYO1B	MYO1B	Myosin IB		97	
PON3	PON3	Paraoxonase 3	300	127	96
RASIP1	RASIP1	Ras interacting protein 1	229		
SDPR	SDPR	Serum deprivation-response protein	575	75	168
SEPT4	SEPT4	Septin 4	88		
SFTPA1	SFTPA1	Surfactant, pulmonary-associated protein A	319	75	466
SFTPB	SFTPB	Surfactant, pulmonary-associated protein B	75		156
SFTPC	SFTPC	Surfactant, pulmonary-associated protein C	294		
SLC6A14	SLC6A14	Solute carrier family 6 member 14	135		75
SLCO2A1	SLCO2A1	Solute carrier organic anion transporter family, member 2A1	120		
SYNE2	SYNE2	Spectrin repeat containing, nuclear envelope 2	82		
THBD	THBD	Thrombomodulin	211	163	

*Proteins identified by PCT extraction.

**Figure 5:**
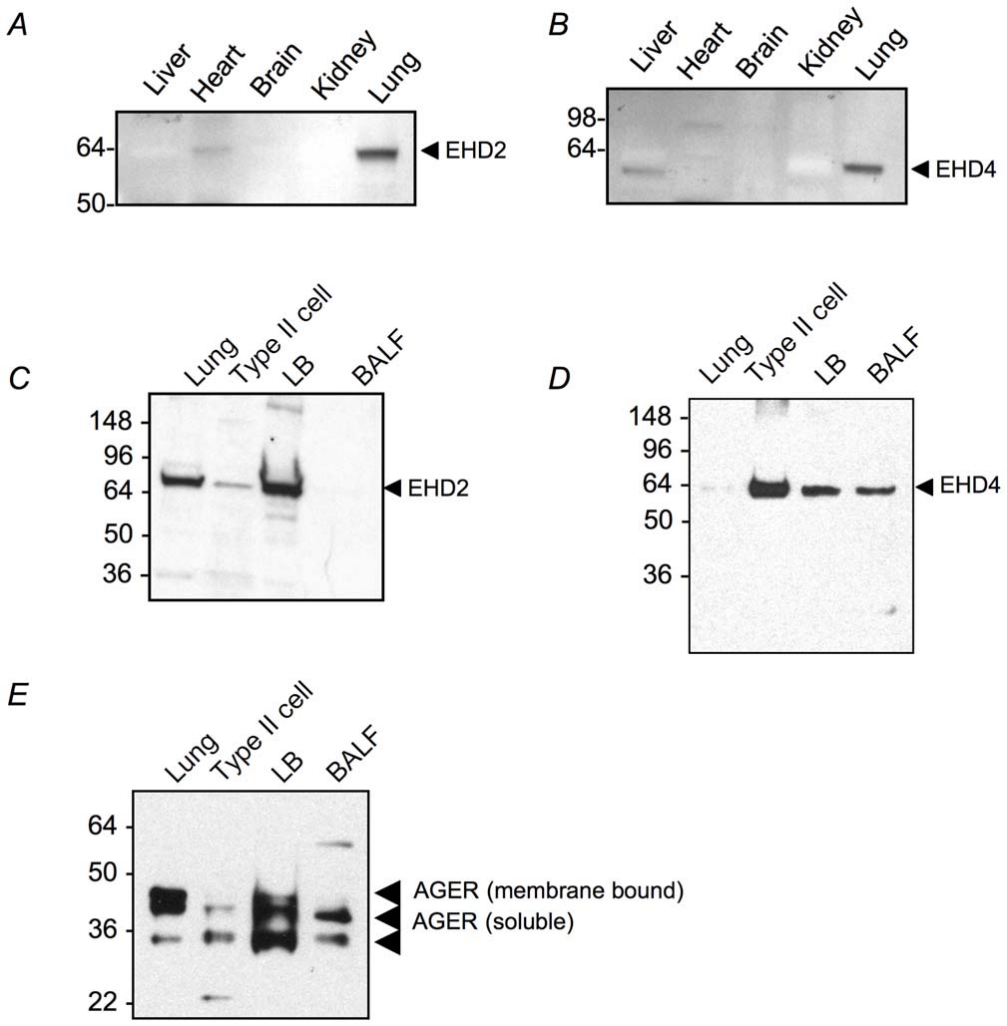
Western blot for LB proteome proteins. Samples were normalized to protein content. Distribution of (A) EHD2 and (B) EHD4 in mouse tissues. Distribution of (C) EHD2, (D) EHD4 and (E) AGER in lung homogenates, type II cells, isolated LBs and BALF.

Pulmonary surfactant is actively recycled from the alveolar spaces likely resulting in the incorporation of some endosomal proteins in the LB. Consistent with this hypothesis a number of endocytic pathway related proteins were identified, including adaptor-related proteins AP2A2 (Mascot score  = 589), and AP2B1 (Mascot score  = 356), clathrin (CLTC, Mascot score  = 1798) and annexins A1, A2 and A6 (Mascot score  = 254, 565 and 1551 respectively). Three EH-domain (EHD) containing proteins, previously implicated in endocytic trafficking, were also identified in the LME fraction [26], [27]. EHD2 and EHD4 had high Mascot scores (1371 and 1019 respectively) and were strongly detected in mouse lung compared to other tissues by western blotting (Figure 5A and B). Although EHD2 was enriched in isolated LB, the signal was lower in type II cells compared to whole lung lysate, suggesting that EHD2 may be expressed in multiple lung cell types (Figure 5C). In contrast EHD4 was detected predominantly in type II cells with lower expression in the LB fraction, suggesting that EHD4 is distributed among multiple subcellular compartments in the type II cells (Figure 5D). Unexpectedly, EHD4 was also found in the BALF, consistent with release into the airspaces.

Multiple proteins involved in airway defense were detected in the LB proteome including antimicrobial and antioxidant proteins. Previously identified proteins in this category included surfactant protein A (Mascot score  = 466) and lysozyme (Mascot score  = 232). Defense proteins not previously detected in the LB include the paraoxonase proteins PON2 and PON3 (Mascot scores 115 and 300 respectively), which are reported to have both anti-oxidant and antimicrobial activities [28]. In addition the antioxidant proteins dolichyl-diphosphooligosaccharide-protein glycosyltransferase (DDOST, Mascot score  = 279) and advanced glycosylation end product-specific receptor (AGER, Mascot score  = 179) were also detected. AGER is highly expressed in the lung but has not previously been associated with the LB. Both membrane (Mw∼50 kDa) and soluble (Mw∼45 kDa) forms of AGER were detected by western blotting of lung homogenates (Figure 5E). Interestingly, soluble AGER was significantly enriched in the LB and was detected in BALF, suggesting that the type II cells may be an important source of this anti-oxidant molecule. The identity of the other AGER isoform (Mw∼34 kDa) has not been established but may represent unglycosylated soluble isoform [29]. Collectively these findings support the conclusion that, in addition to packaging/secretion of pulmonary surfactant, the LB plays an important role in airway defense.

## Discussion

For this study, LBs were isolated from rat lung homogenates by flotation on a discontinuous sucrose gradient. This procedure isolated largely intact organelles as indicated by TEM analysis of LB morphology, significant enrichment of known core LB proteins (SP-C, SP-B, napsin A, lysozyme) and distribution of the limiting membrane protein ABCA3 to the LME fraction and SP-B to the LMD fraction. Initial attempts to digest and identify proteins directly by single LC-MS analyses of isolated intact LBs resulted in the detection of primarily actin peptides, consistent with the previously reported actin mesh surrounding LBs [25], [30]. Sodium carbonate treatment greatly reduced the actin signal and, coupled with SDS-PAGE and the gel gridding method described in the methods section, permitted detection of a total of 468 non-redundant proteins in the intact LB, LME, and LMD fractions (Table S1). Further extraction of sodium carbonate-treated, intact LBs by pressure cycling technology (Table S3) resulted in detection of another 94 proteins, bringing the total number of unique identities to 562. This number is almost certainly an underestimation as relatively few Rab proteins were detected and preliminary western blot analyses of the sodium carbonate, actin-rich fraction identified several proteins not detected by MS but likely to be associated with the LB limiting membrane (e.g., StarD10, data not shown).

Proteins categorized as mitochondrial, nuclear or Golgi comprised ≤6% of the LB proteome, consistent with minor contamination from these subcellular compartments. The fraction of proteins classified as cytosolic (∼14%) was quite similar to that recently reported for LBs isolated from skin (∼18%) [31]. ER proteins comprised ∼13% of the lung LB proteome, a finding that is similar to the range reported for other LROs (11–27%) [23]. Lysosomal, vesicle, plasma membrane and cytoskeletal proteins comprised 59–65% of the proteome consistent with the lysosomal nature of the lamellar body and extensive communication of this organelle with the endocytic and secretory pathways in the type II epithelial cells. Although the dynamic interaction of these organelles complicates assignment of individual proteins to the LB proteome or as contaminants, this broad compartmental overlap is shared by other secretory LROs [23].

The distinguishing feature of LBs is the high content of lipids that are packaged as membrane lamellae. To date, there is only one study of the lung LB proteome that identified 44 proteins in isolated rat lung LBs [15]. Thirty-three of these proteins were detected in the current study and none of the other 11 proteins were unique to the LB (Table 1). The discrepancy in the number of LB proteins identified in the current study (562) and the report by Wang, *et al.* [15] is likely related to different MS procedures employed in the two studies. Consistent with a relatively large number of proteins in the LB proteome, a recent study of LBs isolated from human skin identified 984 proteins [31]. Comparison of the lung and skin LB proteomes revealed a surprisingly small number of shared proteins (9.8%). This finding likely reflects the functional specialization of the two LB populations: skin LBs store lipids primarily involved in barrier function, proteases and glycosidases related to desquamation, and antimicrobial peptides; lung LBs store phospholipids required for surface tension reduction in the alveolar spaces, proteins essential for the organization of intracellular and extracellular surfactant membranes, and distinct proteins involved in innate host defense. Proteins shared by skin and lung LBs were largely associated with or components of the LB limiting membrane, including proteins involved in ion transport or vesicle trafficking.

The lung LB proteome was compared to proteomes of 7 other LROs, recently reviewed by Hu, *et al*. [23], including lysosomes, endosomes, melanosomes, neuromelanin granules, exosomes, platelets, and synaptosomes. Unexpectedly, lung LBs were found to be most similar to melanosomes (37.8% shared proteins). Furthermore, hypergeometric distribution analyses of the LB proteome with human GO annotation (Table S4) identified the melanosome as the compartment most similar to the LB (P = 2.5×10^−30^). Melanosomes are LROs specialized for the synthesis and storage of the pigment melanin. As for skin LBs, many proteins shared by melanosomes and lung LBs were associated with the limiting membranes of these organelles; however, the extent of overlap between lung LB and melanosome was much greater than that for lung and skin LBs. Comparison of the LB proteome with the proteomes for early (immature) and late (mature) melanosomes revealed greater overlap between the LB and early melanosome (Table S5). Collectively, these findings suggest that there is considerable overlap in the biogenesis of LBs and melanosomes. This conclusion is supported by the observations that mutations that disrupt melanosome biogenesis are frequently associated with both hypo-pigmentation disorders and pulmonary fibrosis in patients with Hermansky Pudlak Syndrome (HPS) [32] and lamellar body structure and/or surfactant secretion is affected in several different HPS mouse mutants [33]–[37].

To identify proteins that contribute to the specialized functions of lung LBs, we compared the 9 LRO proteomes and selected the 229 proteins that were unique to the LB proteome. These data were subsequently filtered to identify proteins with confirmed expression in type II epithelial cells (by comparing the unique protein subset to archived microarrays of mouse type II epithelial cells) and elevated expression in lung compared to other tissues. This approach identified 35 proteins, including 4 proteins generally recognized as LB constituents (SP-A, SP-B, SP-C, and ABCA3) (Table 1); three other proteins typically regarded as LB constituents (SP-D, napsin A, and lysozyme) were also present in the lysosome, exosome and skin LB proteomes respectively and thus were not included in this subset.

In addition to ABCA3, 2 potential lipid transport proteins (ABCA8a and ATP8A1) were detected in the LB unique subset of proteins. ABCA8a is a member of the ABCA subfamily of ABC transporters and shows extensive similarity to ABCA5 (59%), ABCA6 (69%), ABCA9 (77%), and ABCA10 (73%), all of which are expressed in brain. Consistent with localization of family members to lipid-rich tissues, ABCA8a was previously shown to transport the lipophilic substrates digoxin and leukotriene C4 [38], [39]. Whether ABCA8a functions as a phospholipid transport protein at the LB membrane remains to be tested. ATP8A1/ATPase II facilitates ATP-dependent transport of phosphatidylserine (PS) across vesicle membranes *in vitro* and has been implicated as the “flippase” that maintains PS asymmetry in the plasma membrane [40], [41]. The yeast homolog of ATP8A1, Drs2p, is localized to the Golgi through interaction with an accessory protein, Cdc50p [42]. The accessory protein may influence both the intracellular localization and substrate preference of the transporter complex. Assuming a membrane orientation similar to the plasma membrane flippase (i.e. such that phospholipid is transferred to the cytosolic leaflet), one possible function for ATP8a1 would be to “recondition” surfactant internalized via the recycling pathway by removing undesirable phospholipid components such as lysoPC from the LB for transport back to the ER. Overall, very little is known about the function of ATP8A1 and ABCA8a and the role of these potential phospholipid transport proteins in the formation and/or remodeling of surfactant membranes.

Several other proteins with lipid-related functions were detected in the LB unique subset, including the prostaglandin transporter SLCO2A1, the myelin associated protein periaxin (PRX), a fatty acid binding protein (Fabp1), a phospholipid binding protein (SDPR), and carboxylesterase 3 (CES3). These proteins have not previously been localized to the LB and, with the possible exception of CES3, their function in this compartment is not known. Carboxylesterase converts large aggregate forms of surfactant to smaller vesicular forms *in vitro* and is postulated to play a role in metabolism of surfactant in the airspaces. Previous studies detected carboxylesterase activity in bronchoalveolar lavage fluid from rats [43] and mice [44], [45] that was identified as CES1 (liver carboxylestrase 1/ES-2), an enzyme that is 72% identical to CES3. CES1 was not detected in the LB proteome and thus the cellular source of this enzyme in the airspaces is unclear. Whether CES3 and/or CES1 are part of a redundant system required for turnover of the alveolar surfactant pool remains to be determined.

The LB unique subset also contained several proteins that may contribute to antioxidant function in the lung, including PON3, ceruloplasmin, glutathione S-transferase and AGER/RAGE. The identification of RAGE in the LB proteome was unexpected as this protein has been reported to be a marker for type I alveolar epithelial cells [29]. Western blotting detected predominantly the soluble isoform of RAGE in both isolated LBs and BALF. Although we cannot exclude the possibility that RAGE is a contaminant, we note that other type I epithelial cell markers (e.g. T1α and aquaporin 5) were not detected in the LB proteome; further, there are conflicting reports regarding the expression pattern of RAGE in the lung [46], [47]. Thus, it is possible that RAGE is synthesized and secreted by type II epithelial cells or internalized into LBs from the airspaces via the endocytic pathway.

In summary we have identified 562 proteins in isolated rat lung LBs. This proteome reflects a highly dynamic organelle that communicates extensively with the biosynthetic, secretory and endocytic pathways. The lung LB proteome significantly overlaps other LRO proteomes and shows striking similarity to the melanosome with surprisingly little similarity to skin LBs. Proteins with lipid-related functions comprise a significant proportion of the LB unique protein subset, consistent with the major function of this organelle in the organization, storage and secretion of surfactant membranes. Although the molecular pathways involved in maturation of the LB remains a significant knowledge gap, identification of multiple phospholipid transport proteins and ER proteins in the LB proteome provide further support for a model involving non-vesicular transfer of phospholipids between the ER and LB. Finally, in addition to proteins that may modulate organization of extracellular surfactant membranes, the lung contains proteins with antimicrobial and antioxidant properties that likely contribute to alveolar homeostasis.

## Supporting Information

Figure S1 Fraction scheme for isolation of limiting membrane proteins from lamellar body.
(TIF)

Figure S2 Immunogold localization for calnexin in (A) isolated rat LB. Gold particles are proximal to lamellar body limiting membrane. Scale bar  = 250 nm. (B) Double label confocal microscopy of mouse lung tissue. Anti-SP-B (green) and anti-PDIA3 (red) colocalize at the lamellar bodies in type II cells. Scale bar  = 10 µm.
(TIF)

Table S1 Rat lamellar body proteome organized by predicted localization to the limiting membrane or core (lumen) of the LB. Proteins detected in two or more fractions (i.e. LB, LME or LMD) received more than one Mascot score. In some cases, one the Mascot scores was below the threshold (indicated by *); at least one Mascot score had to be >75 in order for the protein to be included in the proteome.
(DOC)

Table S2 Subcellular localization of rat LB proteins selected from the gene ontology database (http://www.ebi.ac.uk/GOA/).
(DOC)

Table S3 LB proteins detected only by PCT extraction of the LB sample.
(DOC)

Table S4 The LB proteome was analyzed for subcellular compartment enrichment by hypergeometric distribution using GO annotations of the human genome as reference background [48]. The Benjamini-Hochberg procedure was used to control family-wide false discovery rate ≤0.001 [49]. From the proteins identified in the current LB proteomics study, 16.7% (85 proteins) are known ER residents. The overlap of the LB and ER proteins is significant (P = 1.2×10^−14^). Gene Ontology Analysis was performed using public available web-based tool DAVID [48]. Overrepresented cellular components were selected at the threshold of False Discovery Rate (FDR) ≤0.005, minimum gene counts belonging to an annotation term ≥3% and Fold enrichment >2. Hypergeometric distribution was used to determine the degree of enrichment (PValue), FDR was determined using Benjamini-Hochberg procedure [49]. Fold enrichment measures the magnitude of enrichment, i.e. the % of proteins in LB list with a certain GO term versus the % of proteins with the same GO term in human genome (background).
(DOC)

Table S5 Comparison of LROs and LB proteome.
(DOC)
