# Supplementary figures and images for: Comparative Proteomic Analysis of Lung Lamellar Bodies and Lysosome-Related Organelles
Source: PLoS One. 2011 Jan 26;6(1):e16482. doi: 10.1371/journal.pone.0016482 (PMC3027677; doi:10.1371/journal.pone.0016482)

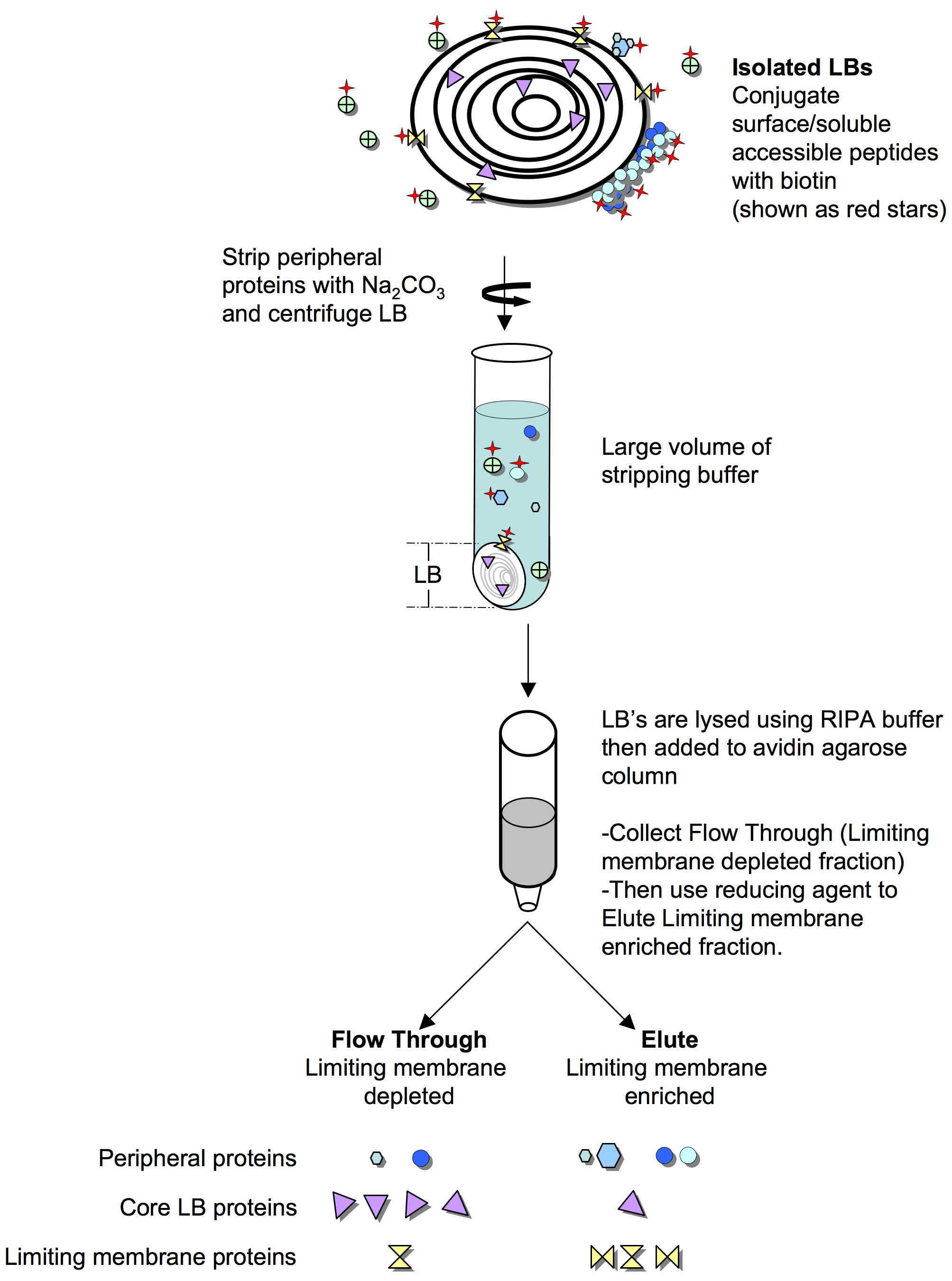

Supplement: Figure S1 — Fraction scheme for isolation of limiting membrane proteins from lamellar body. (TIF) [file pone.0016482.s001.tif]

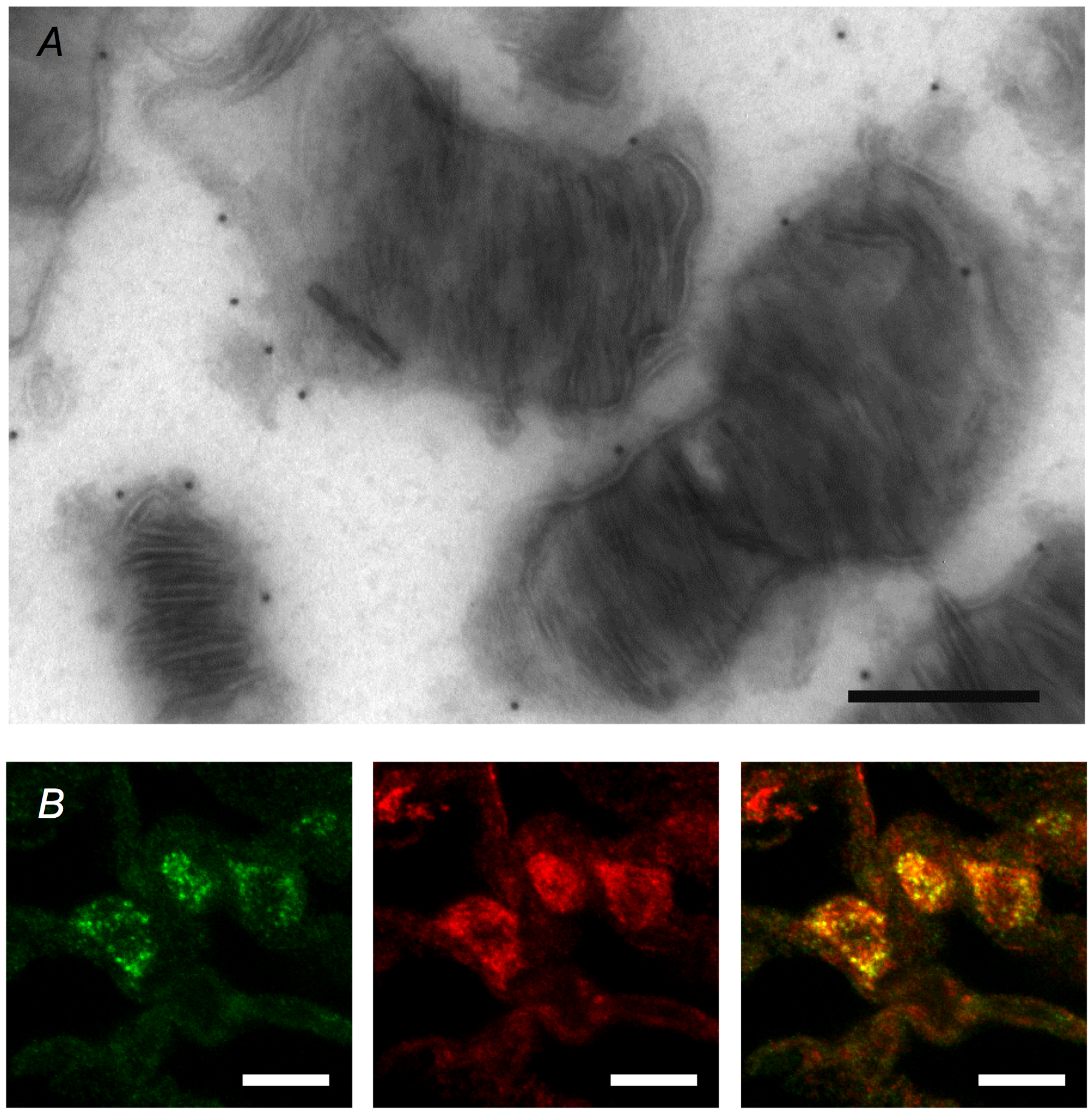

Supplement: Figure S2 — Immunogold localization for calnexin in (A) isolated rat LB. Gold particles are proximal to lamellar body limiting membrane. Scale bar = 250 nm. (B) Double label confocal microscopy of mouse lung tissue. Anti-SP-B (green) and anti-PDIA3 (red) colocalize at the lamellar bodies in type II cells. Scale bar = 10 µm. (TIF) [file pone.0016482.s002.tif]
