# Supplementary material for: Comparative Proteomic Analysis of Lung Lamellar Bodies and Lysosome-Related Organelles
Source: PLoS One. 2011 Jan 26;6(1):e16482. doi: 10.1371/journal.pone.0016482 (PMC3027677; doi:10.1371/journal.pone.0016482)
Supplement: Table S1 — Rat lamellar body proteome organized by predicted localization to the limiting membrane or core (lumen) of the LB. Proteins detected in two or more fractions (i.e. LB, LME or LMD) received more than one Mascot score. In some cases, one the Mascot scores was below the threshold (indicated by *); at least one Mascot score had to be >75 in order for the protein to be included in the proteome. (DOC) [file pone.0016482.s003.doc]

|  |  | Limiting Membrane Proteins |  |  |  | |
| --- | --- | --- | --- | --- | --- | --- |
|  |  |  | Mascot Score | | |  |
| Rat Symbol | Human Symbol | NAME | LB | LME | LMD | |
| ABCA3 | ABCA3 | ATP-binding cassette, sub-family A (ABC1), member 3 | 272 | 673 | 120 | |
| ABCA8A | ABCA8A | ATP-binding cassette sub-family A member 8-A |  | 107 |  | |
| ACE | ACE | Angiotensin I converting enzyme | 1155 | 561 | 233 | |
| ACTN4 | ACTN4 | Actinin, alpha 4 |  | 463 | 369 | |
| ACTR2 | ACTR2 | Actin-related protein 2 | 118 | 364 |  | |
| AGER | AGER | Advanced glycosylation end product-specific receptor | 179 | 123 |  | |
| AK1 | AK1 | Adenylate kinase 1 |  | 76 |  | |
| AKAP1 | AKAP1 | A kinase (PRKA) anchor protein 1 |  | 208 |  | |
| ALB | ALB | Albumin | 1807 | 790 | 337 | |
| ALDH3B1 | ALDH3B1 | Aldehyde dehydrogenase 3 family, member B1 | 158 | 164 |  | |
| ALDOAL1 | ALDOA | Aldolase A, fructose-bisphosphate | 263 | 124 |  | |
| ALPL | ALPL | Alkaline phosphatase | 214 | * |  | |
| ANPEP | ANPEP | Alanyl (membrane) aminopeptidase | 956 | 956 |  | |
| ANXA3 | ANXA3 | Annexin A3 |  | 195 | 163 | |
| ANXA4 | ANXA4 | Annexin A4 | 434 | * |  | |
| ANXA6 | ANXA6 | Annexin A6 | 1551 | 496 | 390 | |
| AOC3 | AOC3 | Amine oxidase, copper containing 3 | 584 | 162 |  | |
| AP2B1 | AP2B1 | Adaptor-related protein complex 2, beta 1 | 356 | 86 |  | |
| ARHGDIA | ARHGDIA | Rho GDP dissociation inhibitor (GDI) alpha |  | 75 |  | |
| ATP1A1 | ATP1A1 | ATPase, Na+/K+ transporting, alpha 1 | 348 | 672 | 168 | |
| ATP2B1 | ATP2B1 | ATPase, Ca++ transporting, plasma membrane 1 | 304 | 233 |  | |
| ATP2B4 | ATP2B4 | ATPase, Ca++ transporting, plasma membrane 4 | 364 | 163 |  | |
| ATP2C2 | ATP2C2 | ATPase, Ca++ transporting, type 2C, member 2 |  | 108 |  | |
| ATP5A1 | ATP5A1 | ATP synthase, H+ transporting, mitochondrial F1 complex, alpha subunit 1, | 197 | 105 |  | |
| ATP5J | ATP5J | ATP synthase, H+ transporting, mitochondrial F0 complex, subunit F6 |  | 75 |  | |
| ATP8A1 | ATP8A1 | ATPase, aminophospholipid transporter (APLT), Class I, type 8A, member 1 | 163 | 119 |  | |
| BCAM | BCAM | Basal cell adhesion molecule | 181 | 148 |  | |
| CANX | CANX | Calnexin | 431 | 438 | 125 | |
| CD34 | CD34 | CD34 molecule | 153 | 82 |  | |
| CD59 | CD59 | CD59 antigen |  | 81 |  | |
| CD9 | CD9 | CD9 antigen | 75 | 91 |  | |
| CDC42 | CDC42 | Cell division cycle 42 (GTP binding protein, 25kDa) | 104 | 131 | 85 | |
| CFL1 | CFL1 | Cofilin 1 |  | 123 |  | |
| CKAP4 | CKAP4 | Cytoskeleton-associated protein 4 | 694 | 93 |  | |
| CKB | CKB | Creatine kinase B-type | 111 | * |  | |
| CLTCL1 | CLTCL1 | Clathrin, heavy chain-like 1 | 360 | 75 |  | |
| COL1A2 | COL1A2 | Collagen, type I, alpha 2, | 88 | * |  | |
| CPM | CPM | Carboxypeptidase M | 139 | * |  | |
| CR1 | CR1 | Complement component (3b/4b) receptor 1 |  | 91 |  | |
| CRIP2 | CRIP2 | Cysteine-rich protein 2 |  | 75 |  | |
| CYB5R2 | CYB5R2 | NADH-cytochrome b5 reductase 2 |  | 75 |  | |
| CYB5R3 | CYB5R3 | Cytochrome b5 reductase 3 | 291 | 75 |  | |
| CYP2B6 | CYP2B6 | Cytochrome P450, family 2, subfamily B, polypeptide 6 |  | 500 |  | |
| CYP2D6 | CYP2D6 | Cytochrome P450, family 2, subfamily D, polypeptide 6 |  | 86 |  | |
| DDB1 | DDB1 | Damage-specific DNA binding protein 1, |  | 75 |  | |
| DNPEP | DNPEP | Aspartyl aminopeptidase |  | 75 |  | |
| DPEP1 | DPEP1 | Dipeptidase 1 | 293 | 270 |  | |
| DPP4 | DPP4 | Dipeptidyl-peptidase 4 | 1034 | 323 |  | |
| DSTN | DSTN | Destrin |  | 75 |  | |
| ECE1 | ECE1 | Endothelin converting enzyme 1 | 85 | 263 |  | |
| EFHD2 | EFHD2 | EF-hand domain family, member D2 |  | 75 |  | |
| EHD1 | EHD1 | EH-domain containing 1 | 447 | 221 |  | |
| EHD2 | EHD2 | EH-domain containing 2 | 1371 | 447 | 384 | |
| EHD4 | EHD4 | EH-domain containing 4 | 1019 | * |  | |
| EIF2C3 | EIF2C3 | Eukaryotic translation initiation factor 2C, 3 |  | 75 |  | |
| EIF5A | EIF5A | Eukaryotic translation initiation factor 5A |  | 75 |  | |
| ENO1 | ENO1 | Enolase 1 | 105 | 75 |  | |
| ENPEP | ENPEP | Glutamyl aminopeptidase | 500 | 261 |  | |
| ESAM | ESAM | Endothelial cell adhesion molecule |  | 101 |  | |
| FCRLS | FCRL2 | Fc receptor-like 2 |  | 156 |  | |
| FLNA | FLNA | Filamin A, alpha |  | 75 |  | |
| FMR1 | FMR1 | Fragile X mental retardation 1 protein |  | 75 |  | |
| GNA11 | GNA11 | Guanine nucleotide-binding protein subunit alpha-11 | 166 | 166 |  | |
| GNAI3 | GNAI3 | Guanine nucleotide binding protein (G protein), alpha inhibiting activity polypeptide 3 | 416 | 89 |  | |
| GNAQ | GNAQ | Guanine nucleotide binding protein (G protein), q polypeptide | 318 | 187 |  | |
| GNAS | GNAS | Guanine nucleotide-binding protein G(s) subunit alpha | 235 | 116 |  | |
| GNAT1 | GNAT1 | Guanine nucleotide binding protein (G protein), alpha transducing activity polypeptide 1 |  | 78 |  | |
| GNAT3 | GNAT3 | Guanine nucleotide binding protein (G protein), alpha transducing activity polypeptide 3 | 201 | 548 |  | |
| GNB1 | GNB1 | Guanine nucleotide binding protein (G protein), beta polypeptide 1 | 328 | 346 |  | |
| GNB2 | GNB2 | Guanine nucleotide binding protein (G protein), beta polypeptide 2 | 427 | 307 |  | |
| GPIAP1 | GPIAP1 | GPI-anchored membrane protein 1 |  | 75 |  | |
| GPRC5A | GPRC5A | G protein-coupled receptor, family C, group 5, member A | 204 | * |  | |
| GSTA3 | GSTA3 | Glutathione S-transferase A3 |  | 75 |  | |
| GTF2H4 | GTF2H4 | General transcription factor IIH, polypeptide 4 |  | 75 |  | |
| GUSB | GUSB | Glucuronidase, beta |  | 75 |  | |
| HBA | HBA | Hemoglobin alpha-2 chain |  | 138 |  | |
| HNRNPR | HNRNPR | Heterogeneous nuclear ribonucleoprotein R |  | 75 |  | |
| HNRPA3 | HNRPA3 | Heterogeneous nuclear ribonucleoprotein A3 |  | 75 |  | |
| HSPB1 | HSPB1 | Heat shock protein beta-1 |  | 75 |  | |
| ICAM1 | ICAM1 | Intercellular adhesion molecule 1 | 103 | * |  | |
| IGSF4D | IGSF4D | Cell adhesion molecule 2 |  | 90 |  | |
| ITGA3 | ITGA3 | Integrin, alpha 3 |  | 94 |  | |
| ITGAV | ITGAV | Integrin, alpha V |  | 100 |  | |
| ITGB1 | ITGB1 | Integrin beta 1 | 544 | 264 |  | |
| KB1 | KRT1 | Keratin | 118 | 75 |  | |
| KCTD12 | KCTD12 | Potassium channel tetramerisation domain containing 12 | 103 | 75 |  | |
| KTN1 | KTN1 | Kinectin 1 | 85 | * |  | |
| LMO7 | LMO7 | LIM domain 7 |  | 75 |  | |
| LOC286911 | PRSS3 | Trypsin | 104 | * |  | |
| LOC691499 | ZNF469 | Zinc finger protein 469 | 79 | * |  | |
| LRP1 | LRP1 | Low density lipoprotein-related protein 1 |  | 108 |  | |
| LRP2 | LRP2 | Low density lipoprotein-related protein 2 | 94 | 78 |  | |
| LRRFIP2 | LRRFIP2 | Leucine rich repeat (in FLII) interacting protein 2 | 78 | * |  | |
| MBC2 | FAM62A | Family with sequence similarity 62 (C2 domain containing), member A | 557 | 77 |  | |
| MGC72560 | C11ORF59 | Chromosome 11 open reading frame 59 |  | 78 |  | |
| MGST1 | MGST1 | Microsomal glutathione S-transferase 1 |  | 115 |  | |
| MYH10 | MYH10 | Myosin, heavy chain 10 | 447 | 250 |  | |
| MYH9 | MYH9 | Myosin, heavy polypeptide 9, non-muscle |  | 94 |  | |
| MYL6 | MYL6 | Myosin, light chain 6, alkali, smooth muscle and non-muscle |  | 117 |  | |
| MYL9 | MYL9 | Myosin, light chain 9, regulatory |  | 84 |  | |
| MYO1B | MYO1B | Myosin IB |  | 97 |  | |
| NDUFV1 | NDUFV1 | NADH dehydrogenase (ubiquinone) flavoprotein 1 |  | 75 |  | |
| PDIA6 | PDIA6 | Protein disulfide isomerase associated 6 |  | 105 |  | |
| PFN1 | PFN1 | Profilin 1 |  | 75 |  | |
| PGRMC1 | PGRMC1 | Progesterone receptor membrane component 1 |  | 179 |  | |
| PHB | PHB | Prohibitin |  | 89 |  | |
| PLVAP | PLVAP | Plasmalemma vesicle associated protein | 285 | * |  | |
| PON2 | PON2 | Paraoxonase 2 | 115 | 79 |  | |
| PON3 | PON3 | Paraoxonase 3 | 300 | 127 | 96 | |
| PPIA | PPIA | Peptidylprolyl isomerase A (cyclophilin A) |  | 221 | 127 | |
| PPP1R14A | PPP1R14A | Protein phosphatase 1, regulatory (inhibitor) subunit 14A |  | 75 |  | |
| PRDX2 | PRDX2 | Peroxiredoxin 2 |  | 75 |  | |
| PRKAR2A | PRKAR2A | Protein kinase, cAMP-dependent, regulatory, type II, alpha |  | 75 |  | |
| PRX | PRX | Periaxin | 2184 | 746 | 109 | |
| PSME3 | PSME3 | Proteasome (prosome, macropain) activator subunit 3 |  | 75 |  | |
| PTGIS | PTGIS | Prostacyclin synthase |  | 77 |  | |
| PURA | PURA | Purine-rich element binding protein A |  | 75 |  | |
| PURB | PURB | Purine-rich element binding protein B |  | 75 |  | |
| RAC1 | RAC1 | Ras-related C3 botulinum toxin substrate 1 |  | 105 |  | |
| RAP1A | RAP1A | Ras-related protein Rap-1A | 95 | 180 | 86 | |
| RCN1 | RCN1 | Reticulocalbin-1 |  | 75 |  | |
| RCN3 | RCN3 | Reticulocalbin-3 |  | 75 |  | |
| RGD1304704 | C14ORF166 | Chromosome 14 open reading frame 166 |  | 75 |  | |
| RGD1307018 | FAM129B | Family with sequence similarity 129, member B, Niban-like |  | 93 |  | |
| RGD1309871 | ATL3 | Atlastin 3 |  | 96 |  | |
| RGD1310121 | TPPP | Tubulin polymerization promoting protein |  | 75 |  | |
| RGD1311703 | C11ORF58 | Chromosome 11 open reading frame 58 |  | 75 |  | |
| RGD1563977 | EPB41L2 | Erythrocyte membrane protein band 4.1-like 2 | 648 | 171 |  | |
| RGD1564835 | HEPHL1 | Hephaestin-like 1 |  | 277 |  | |
| RHOA | RHOA | Ras homolog gene family, member A | 114 | 179 |  | |
| RHOG | RHOG | Ras homolog gene family, member G |  | 244 | 77 | |
| RPN1 | RPN1 | Ribophorin I | 280 | 130 |  | |
| RRBP1 | RRBP1 | Ribosome binding protein 1 | 151 | * |  | |
| RSAFD1 | LOC650433 | Similar to Radical S-adenosyl methionine and flavodoxin domains 1 |  | 282 |  | |
| RT1-A2 | HLA-A | Major histocompatibility complex, class I, A | 285 | 87 |  | |
| RTN4 | RTN4 | Reticulon 4 | 183 | * |  | |
| S100A11 | S100A11 | S100 calcium binding protein A11 | 75 | * |  | |
| SDF2L1 | SDF2L1 | Stromal cell-derived factor 2-like 1 |  | 127 |  | |
| SEPT7 | SEPT7 | Septin 7 | 292 | * |  | |
| SERPINH1 | SERPINH1 | Serpin peptidase inhibitor, clade H (heat shock protein 47), member 1, | 459 | 102 |  | |
| SLC3A2 | SLC3A2 | Solute carrier family 3 member 2 | 118 | * |  | |
| SLC4A1 | SLC4A1 | Solute carrier family 4 member 1 | 393 | * |  | |
| SPTAN1 | SPTA1 | Spectrin, alpha, erythrocytic 1 |  | 75 |  | |
| SPTBN5 | SPTBN5 | Spectrin, beta, non-erythrocytic 5 | 110 | 75 |  | |
| SUSD2 | SUSD2 | Sushi domain-containing protein 2 | 223 | 189 |  | |
| TAGLN2 | TAGLN2 | Transgelin-2 |  | 75 |  | |
| TF | TF | Transferrin | 664 | 153 |  | |
| THBD | THBD | Thrombomodulin | 211 | 163 |  | |
| TLN1 | TLN1 | Talin 1 | 471 | 458 |  | |
| VAMP3 | VAMP3 | Vesicle-associated membrane protein 3 |  | 111 |  | |
| VAT1 | VAT1 | Vesicle amine transport protein 1 homolog | 377 | 75 |  | |
| XPNPEP2 | XPNPEP2 | X-prolyl aminopeptidase (aminopeptidase P) 2, membrane-bound | 780 | 586 |  | |
| YBX1 | YBX1 | Y box binding protein 1 |  | 75 |  | |
| YWHAH | YWHAH | Tyrosine 3-monooxygenase/tryptophan 5-monooxygenase activation protein, eta |  | 75 |  | |
| YWHAQ | YWHAQ | Tyrosine 3-monooxygenase/tryptophan 5-monooxygenase activation protein, theta |  | 75 |  | |
| YWHAZ | YWHAZ | Tyrosine 3-monooxygenase/tryptophan 5-monooxygenase activation protein, zeta |  | 75 |  | |
| ZYX | ZYX | Zyxin |  | 75 |  | |
|  |  |  |  |  |  | |
|  |  |  |  |  |  | |
|  |  | Core Proteins |  |  |  | |
|  |  |  | Mascot Score | | | |
| Rat Symbol | Human Symbol | NAME | LB | LME | LMD | |
| A2M | A2M | Alpha-2-macroglobulin |  |  | 99 | |
| ACTB | ACTB | Beta-actin | 1252 | 640 | 762 | |
| ANXA1 | ANXA1 | Annexin A1 | 254 | 108 | 200 | |
| ANXA2 | ANXA2 | Annexin A2 | 565 | 203 | 338 | |
| ANXA5 | ANXA5 | Annexin A5 | 406 | 91 | 671 | |
| AOC2 | AOC2 | Amine oxidase, copper containing 2 | 78 |  | 213 | |
| AQP1 | AQP1 | Aquaporin 1 |  |  | 148 | |
| ATP2A2 | ATP2A2 | ATPase, Ca++ transporting, cardiac muscle, slow twitch 2 | 118 | 214 | 297 | |
| ATP5B | ATP5B | ATP synthase, H+ transporting, mitochondrial F1 complex, beta | 242 |  | 146 | |
| BLVRB | BLVRB | Biliverdin reductase B |  |  | 75 | |
| C3 | C3 | Complement component 3 | 129 |  | 178 | |
| CA2 | CA2 | Carbonic anhydrase II |  |  | 86 | |
| CABP1 | CABP1 | Calcium binding protein 1 | 371 |  | 75 | |
| CALM2 | CALM2 | Calmodulin 2 |  | 75 | 137 | |
| CALU | CALU | Calumenin |  | 75 | 93 | |
| CAPN2 | CAPN2 | Calpain 2 |  |  | 245 | |
| CAT | CAT | Catalase | 118 |  | * | |
| CD31 | CD31 | Platelet/endothelial cell adhesion molecule (CD31 antigen) | 137 |  | 105 | |
| CLTC | CLTC | Clathrin, heavy chain | 1798 | 75 | 630 | |
| CP | CP | Ceruloplasmin | 905 |  | 136 | |
| CYP4B1 | CYP4B1 | Cytochrome P450, family 4, subfamily B, polypeptide 1 | 174 | 75 | 177 | |
| CYB5B | CYB5B | cytochrome b5 type B |  | 138 | 157 | |
| DPYSL2 | DPYSL2 | Dihydropyrimidinase-like 2 | 409 |  | 263 | |
| EEF2 | EEF2 | Elongation factor 2 | 204 |  | * | |
| EZR | EZR | Ezrin | 80 | 75 | 150 | |
| FABP4 | FABP4 | Fatty acid binding protein 4 |  | 233 | 258 | |
| GANAB | GANAB | Neutral alpha-glucosidase AB precursor | 244 |  | 88 | |
| GDA | GDA | Guanine deaminase | 414 | 155 | 272 | |
| GSN | GSN | Gelsolin | 347 | 75 | 183 | |
| GSTP1 | GSTP1 | Glutathione S-transferase P |  |  | 181 | |
| HBB | HBB | Hemoglobin |  | 507 | 661 | |
| HNRNPA2B1 | HNRNPA2B1 | Heterogeneous nuclear ribonucleoprotein A2/B1 | 76 | 75 | 95 | |
| HSPA5 | HSPA5 | Heat shock 70kDa protein 5 | 1415 | 550 | 774 | |
| HSPA8 | HSPA8 | Heat shock 70kDa protein 8 | 840 | 75 | 274 | |
| HSPCA | HSP90AA1 | Heat shock protein 90kDa alpha (cytosolic), class A member 1 |  | 75 | 238 | |
| HSPCB | HSP90AB1 | Heat shock protein 90kDa alpha (cytosolic), class B member 1 |  | 75 | 414 | |
| HYOU1 | HYOU1 | Hypoxia up-regulated protein 1 | 214 |  | 98 | |
| IQGAP1 | IQGAP1 | IQ motif containing GTPase activating protein 1 | 1297 | 125 | 150 | |
| LCP1 | LCP1 | Lymphocyte cytosolic protein 1 (L-plastin) | 87 | 75 | 200 | |
| LDHA | LDHA | Lactate dehydrogenase A | 109 |  | 125 | |
| LYN | LYN | v-yes-1 Yamaguchi sarcoma viral related oncogene homolog | 449 | 176 | 210 | |
| LYZ | LYZ | Lysozyme | 199 | 98 | 232 | |
| MARCKS | MARCKS | Myristoylated alanine-rich protein kinase C substrate | 402 | 75 | 148 | |
| MCAM | MCAM | Melanoma cell adhesion molecule | 96 |  | * | |
| MDH1 | MDH1 | Malate dehydrogenase 1 | 78 |  | * | |
| MGC109519 | TPM2 | Tropomyosin 2 |  | 75 | 148 | |
| MSN | MSN | Moesin | 1967 | 75 | 577 | |
| MVP | MVP | Major vault protein | 546 | 75 | 456 | |
| MYH11 | MYH11 | Myosin, heavy chain 11 | 2411 | 114 | 744 | |
| NAPSA | NAPSA | Napsin A aspartic peptidase | 101 |  | 97 | |
| NP | NP | Nnucleoside phosphorylase |  | 75 | 225 | |
| NUCB1 | NUCB1 | Nucleobindin 1 | 141 |  | 89 | |
| P4HB | P4HB | Procollagen-proline, 2-oxoglutarate 4-dioxygenase (proline 4-hydroxylase), beta polypeptide | 443 | 75 | 384 | |
| PDIA3 | PDIA3 | Protein disulfide isomerase family A, member 3 | 882 | 114 | 434 | |
| POR | POR | P450 (cytochrome) oxidoreductase | 494 |  | 111 | |
| PRDX6 | PRDX6 | Peroxiredoxin-6 |  | 75 | 469 | |
| PRKCSH | PRKCSH | Protein kinase C substrate 80K-H | 86 |  | 75 | |
| PTRF | PTRF | Polymerase I and transcript release factor |  | 75 | 97 | |
| PZP | PZP | Pregnancy-zone protein |  |  | 153 | |
| RAB1A | RAB1A | RAB1, member RAS oncogene family |  |  | 115 | |
| RCN2 | RCN2 | Reticulocalbin-2 |  | 75 | 118 | |
| RDX | RDX | Radixin | 707 |  | 83 | |
| RGD1562954 | AKR1C4 | Aldo-keto reductase family 1, member C4 | 85 |  | 129 | |
| RPN2 | RPN2 | Ribophorin II | 234 |  | 209 | |
| SCARB2 | SCARB2 | Scavenger receptor class B, member 2 | 243 |  | 129 | |
| SCPEP1 | SCPEP1 | Serine carboxypeptidase 1 |  |  | 92 | |
| SDPR | SDPR | Serum deprivation-response protein | 575 | 75 | 168 | |
| SEC14L3 | SEC14L3 | SEC14-like 3 | 270 | 75 | 136 | |
| SERPINA3N | SERPINA3 | Serpin peptidase inhibitor, clade A (alpha-1 antiproteinase, antitrypsin), member 3 | 130 |  | 98 | |
| SERPINB6 | SERPINB6 | Serine (or cysteine) peptidase inhibitor, clade B, member 6a | 113 |  | 91 | |
| SFTPA1 | SFTPA1 | Surfactant, pulmonary-associated protein A | 319 | 75 | 466 | |
| SFTPB | SFTPB | Surfactant, pulmonary-associated protein B | 75 |  | 156 | |
| SFTPC | SFTPC | Surfactant, pulmonary-associated protein C | 294 |  | * | |
| SH3BGRL3 | SH3BGRL3 | SH3 domain binding glutamic acid-rich protein like 3 |  |  | 82 | |
| SLC6A14 | SLC6A14 | Solute carrier family 6 member 14 | 135 |  | 75 | |
| SOD1 | SOD1 | Superoxide dismutase 1 |  |  | 269 | |
| SPNA2 | SPTAN1 | Spectrin, alpha, non-erythrocytic 1 |  |  | 98 | |
| TKT | TKT | Transketolase | 253 | 75 | 254 | |
| TMED10 | TMED10 | Transmembrane emp24-like trafficking protein 10 | 98 |  | * | |
| TMEM43 | TMEM43 | Transmembrane protein 43 | 137 |  | * | |
| TPM1 | TPM1 | Tropomyosin 1 |  | 75 | 164 | |
| TRA1 | HSP90B1 | Heat shock protein 90kDa beta (Grp94), member 1 | 881 | 89 | 416 | |
| TXNDC4 | TXNDC4 | Thioredoxin domain-containing protein 4 | 139 |  | 254 | |
| UBE1X | UBE1 | Ubiquitin-like modifier activating enzyme 1 | 125 |  | * | |
| VCL | VCL | Vinculin | 418 | 75 | 166 | |
| VCP | VCP | Valosin-containing protein | 371 | 75 | 233 | |
| YWHAE | YWHAE | Tyrosine 3-monooxygenase/tryptophan 5-monooxygenase activation protein, epsilon | 153 | 75 | 197 | |
|  |  |  |  |  |  | |
|  |  |  |  |  |  | |
|  |  | Not assigned to a compartment |  |  |  | |
|  |  |  | Mascot Score | | | |
| Rat Symbol | Human Symbol | NAME | LB | LME | LMD | |
| ACACA | ACACA | Acetyl-CoA carboxylase 1 | 85 |  |  | |
| ACTA1 | ACTA1 | Actin, alpha 1, skeletal muscle | 662 |  |  | |
| ACTG1 | ACTG1 | Actin, gamma 1 | 1102 |  |  | |
| ADAM10 | ADAM10 | ADAM metallopeptidase domain 10 | 77 |  |  | |
| AKAP5 | AKAP5 | A kinase (PRKA) anchor protein 5 | 136 |  |  | |
| ALCAM | ALCAM | Activated leukocyte cell adhesion molecule | 161 |  |  | |
| ALDH3A2 | ALDH3A2 | Aldehyde dehydrogenase 3 family, member A2 | 197 |  |  | |
| ALDOC | ALDOC | Aldolase C, fructose-bisphosphate | 175 |  |  | |
| ANKRD7 | POTE2 | ANKRD26-like family C, member 1A | 480 |  |  | |
| ANXA11 | ANXA11 | Annexin A11 | 92 |  |  | |
| AP2A1 | AP2A1 | Adaptor-related protein complex 2, alpha 1 | 332 |  |  | |
| AP2A2 | AP2A2 | Adaptor-related protein complex 2, alpha 2 | 589 |  |  | |
| AP2M1 | AP2M1 | Adaptor-related protein complex 2, mu 1 | 116 |  |  | |
| APC | APC | Adenomatosis polyposis coli | 108 |  |  | |
| APOE | APOE | Apolipoprotein E | 167 |  |  | |
| APOH | APOH | Apolipoprotein H | 93 |  |  | |
| APP | APP | Amyloid beta (A4) precursor protein | 92 |  |  | |
| ARHGAP23 | ARHGAP23 | Rho GTPase activating protein 23 | 85 |  |  | |
| ARHGEF5 | ARHGEF5 | Rho guanine nucleotide exchange factor (GEF) 5 | 81 |  |  | |
| ARTS1 | ARTS1 | Type 1 tumor necrosis factor receptor shedding aminopeptidase regulator | 121 |  |  | |
| ATP1A3 | ATP1A3 | ATPase, Na+/K+ transporting, alpha 3 | 75 |  |  | |
| ATP2B2 | ATP2B2 | ATPase, Ca++ transporting, plasma membrane 2 | 109 |  |  | |
| ATP6V0A1 | ATP6V0A1 | ATPase, H+ transporting, lysosomal V0 subunit a1 | 199 |  |  | |
| ATP6V1B2 | ATP6V1B2 | ATPase, H+ transporting, lysosomal 56/58kDa, V1 subunit B2 | 77 |  |  | |
| AYTL2 | AYTL2 | Acyltransferase like 2 | 107 |  |  | |
| BLK | BLK | B lymphoid tyrosine kinase | 75 |  |  | |
| BRCA1 | BRCA1 | Breast cancer 1, early onset | 109 |  |  | |
| CA4 | CA4 | Carbonic anhydrase IV | 206 |  |  | |
| CALR | CALR | Calreticulin | 212 |  |  | |
| CAP350 | CAP350 | Centrosome-associated protein 350 | 97 |  |  | |
| CATNA1 | CATNA1 | Alpha(E)-catenin | 433 |  |  | |
| CAV | CAV1 | Caveolin 1 | 122 | 113 | 116 | |
| CD36 | CD36 | CD36 molecule | 283 |  |  | |
| CDC37 | CDC37 | Cell division cycle 37 homolog | 108 |  |  | |
| CDH13 | CDH13 | Cadherin 13 | 82 |  |  | |
| CDIPT | CDIPT | CDP-diacylglycerol--inositol 3-phosphatidyltransferase | 93 |  |  | |
| CENPF | CENPF | Centromere protein F | 83 |  |  | |
| CENTG1 | CENTG1 | Centaurin-gamma-1 | 75 |  |  | |
| CES3 | CES3 | Carboxylesterase 3 | 556 |  |  | |
| CFB | CFB | Complement factor B | 75 |  |  | |
| CHD9 | CHD9 | chromodomain helicase DNA binding protein 9 | 77 |  |  | |
| CNP1 | CNP1 | 2',3'-cyclic nucleotide 3' phosphodiesterase | 162 |  |  | |
| COL6A3 | COL6A3 | Collagen, type VI, alpha 3 | 86 |  |  | |
| COL9A2 | COL9A2 | Collagen, type IX, alpha 2, | 78 |  |  | |
| COLEC12 | COLEC12 | Collectin-12 | 102 |  |  | |
| CTNNB1 | CTNNB1 | Beta-catenin | 202 |  |  | |
| CYFIP1 | CYFIP1 | Cytoplasmic FMR1 interacting protein 1 | 250 |  |  | |
| CYLD | CYLD | Cylindromatosis | 125 |  |  | |
| CYP2A3A | CYP2A13 | Cytochrome P450, family 2, subfamily A, polypeptide 13 | 75 |  |  | |
| CYP2F2 | CYP2F2 | Cytochrome P450, family 2, subfamily f, polypeptide 2 | 252 |  |  | |
| CYP2F4 | CYP2F1 | Cytochrome P450, family 2, subfamily f, polypeptide 4 | 342 |  |  | |
| CYP2S1 | CYP2S1 | Cytochrome P450, family 2, subfamily S, polypeptide 1 | 139 |  |  | |
| DCHS1 | DCHS1 | Protocadherin-16 | 119 |  |  | |
| DDOST | DDOST | Dolichyl-diphosphooligosaccharide-protein glycosyltransferase | 279 |  |  | |
| DLC1 | DLC1 | Deleted in liver cancer 1 | 75 |  |  | |
| DMD | DMD | Dystrophin | 94 |  |  | |
| DNAH1 | DNAH1 | Dynein, axonemal, heavy chain 1 | 89 |  |  | |
| DNAH10 | DNAH10 | Dynein, Axonemal, heavy chain 10 | 89 |  |  | |
| DOCK6 | DOCK6 | Dedicator of cytokinesis protein 6 | 84 |  |  | |
| DST | DST | Dystonin | 124 |  |  | |
| EEF1A1 | EEF1A1 | Eukaryotic translation elongation factor 1 alpha 1 | 486 |  |  | |
| EEF1A2 | EEF1A2 | Eukaryotic translation elongation factor 1 alpha 1 | 291 |  |  | |
| ENDOD1 | ENDOD1 | Endonuclease domain-containing 1 | 82 |  |  | |
| EPHX1 | EPHX1 | Epoxide hydrolase 1 | 301 |  |  | |
| ERLIN1 | ERLIN1 | ER lipid raft associated 1 | 104 |  |  | |
| ERLIN2 | ERLIN2 | ER lipid raft associated 2 | 194 |  |  | |
| EXOC4 | EXOC4 | Exocyst complex component 4 | 82 |  |  | |
| FAM129A | FAM129A | Family with sequence similarity 129, member A, Niban | 200 |  |  | |
| FASN | FASN | Fatty acid synthase | 103 |  |  | |
| FKBP1A | FKBP1A | FK506 binding protein 1A |  | 75 | 77 | |
| FLOT1 | FLOT1 | Flotillin 1 | 133 |  |  | |
| FLOT2 | FLOT2 | Flotillin 2 | 243 |  |  | |
| FMO1 | FMO1 | Flavin containing monooxygenase 1 | 268 |  |  | |
| GCN1L1 | GCN1L1 | General control of amino-acid synthesis 1-like 1 | 82 |  |  | |
| GCS1 | GCS1 | Glucosidase I | 213 |  |  | |
| GDI2 | GDI2 | Rab GDP dissociation inhibitor beta | 87 |  |  | |
| GLG1 | GLG1 | Golgi apparatus protein 1 | 160 |  |  | |
| GNA13 | GNA13 | Guanine nucleotide binding protein (G protein), alpha 13 | 279 |  |  | |
| GNA14 | GNA14 | Guanine nucleotide binding protein (G protein), alpha 14 | 259 |  |  | |
| GNA15 | GNA15 | Guanine nucleotide binding protein (G protein), alpha 15 | 99 |  |  | |
| GNAI1 | GNAI1 | Guanine nucleotide binding protein (G protein), alpha inhibiting activity polypeptide 1 | 284 |  |  | |
| GNAI2 | GNAI2 | Guanine nucleotide binding protein (G protein), alpha inhibiting activity polypeptide 2 | 783 |  |  | |
| GNAL | GNAL | Guanine nucleotide binding protein (G protein), alpha activating activity polypeptide | 125 |  |  | |
| GNAO1 | GNAO1 | Guanine nucleotide binding protein (G protein), alpha activating activity polypeptide O | 390 |  |  | |
| GNAT2 | GNAT2 | Guanine nucleotide binding protein (G protein), alpha transducing activity polypeptide 2 | 203 |  |  | |
| GNB3 | GNB3 | Guanine nucleotide binding protein (G protein), beta polypeptide 3 | 191 |  |  | |
| HDLBP | HDLBP | High density lipoprotein binding protein (vigilin) | 90 |  |  | |
| HERC1 | HERC1 | Guanine nucleotide exchange factor p532 | 84 |  |  | |
| HSD11B1 | HSD11B1 | Hydroxysteroid (11-beta) dehydrogenase 1 | 191 |  |  | |
| HSPA1A | HSPA1A | Heat shock 70kDa protein 1A | 288 |  |  | |
| HSPA1B | HSPA6 | Heat shock 70kDa protein 6 | 278 |  |  | |
| IMMT | IMMT | Mitochondrial inner membrane protein | 85 |  |  | |
| ITGA1 | ITGA1 | Integrin alpha-1 | 252 |  |  | |
| ITGAL | ITGAL | Integrin alpha-L | 105 |  |  | |
| ITGAM | ITGAM | Integrin alpha-M | 138 |  |  | |
| ITGB2 | ITGB2 | Integrin beta-2 | 115 |  |  | |
| JUP | JUP | Junction plakoglobin | 160 |  |  | |
| KIF15 | KIF15 | Kinesin family member 15 | 79 |  |  | |
| KIF16B | C20ORF23 | Chromosome 20 open reading frame 23 | 93 |  |  | |
| KIF26A | KIF26A | Kinesin family member 26A | 83 |  |  | |
| LAMA5 | LAMA5 | Laminin, alpha 5 | 81 |  |  | |
| LAMP1 | LAMP1 | Lysosomal-associated membrane protein 1 | 135 |  |  | |
| LMAN1L | LMAN1 | Lectin, mannose-binding, 1 | 245 |  |  | |
| LMAN2L | LMAN2 | Lectin, mannose-binding, 2 | 133 |  |  | |
| LNPEP | LNPEP | Leucyl/cystinyl aminopeptidase | 200 |  |  | |
| LOC287867 | CCDC40 | Coiled-coil domain containing 40 | 75 |  |  | |
| LOC299949 | WDR67 | WD repeat domain 67 | 77 |  |  | |
| LOC362587 | MACF1 | Microtubule-actin crosslinking factor 1 | 86 |  |  | |
| LOC363267 | ZFP106 | Zinc finger protein 106 homolog | 80 |  |  | |
| LOC499912 | C20ORF3 | Chromosome 20 open reading frame 3 | 299 |  |  | |
| LOC500363 | TUBA2 | Tubulin, alpha 3d | 203 |  |  | |
| LOC500373 | CHMP4B | Chromatin modifying protein 4B | 154 |  |  | |
| LOC680692 | GOLM1 | Golgi membrane protein 1 | 279 |  |  | |
| LOC686653 | ZNF596 | Zinc finger protein 596 | 92 |  |  | |
| LRRK1 | LRRK1 | Leucine-rich repeat kinase 1 | 95 |  |  | |
| M6PR | M6PR | Mannose-6-phosphate receptor | 234 |  |  | |
| MAP3K1 | MAP3K1 | Mitogen-activated protein kinase kinase kinase 1 | 82 |  |  | |
| METTL7A | METTL7A | Methyltransferase like 7A | 95 |  |  | |
| MGC94145 | C1ORF179 | Chromosome 1 open reading frame 179 | 166 |  |  | |
| MME | MME | Membrane metallo-endopeptidase | 373 |  |  | |
| MRC1 | MRC1 | Mannose receptor, C type 1 | 116 |  |  | |
| MRLCB | MRCL3 | Myosin regulatory light chain MRCL3 | 75 |  |  | |
| MYH14 | MYH14 | Myosin, heavy chain 14 | 1651 |  |  | |
| MYH2 | MYH2 | Myosin, heavy chain 2 | 75 |  |  | |
| MYO1C | MYO1C | Myosin IC | 1247 |  |  | |
| MYO6 | MYO6 | Myosin VI | 246 |  |  | |
| NCOR1 | NCOR1 | Nuclear receptor co-repressor 1 | 76 |  |  | |
| NDUFS1 | NDUFS1 | NADH dehydrogenase (ubiquinone) Fe-S protein 1 | 85 |  |  | |
| NEB | NEB | Nebulin | 96 |  |  | |
| NNT | NNT | Nicotinamide nucleotide transhydrogenase | 76 |  |  | |
| NRIP3 | NRIP3 | Nuclear receptor interacting protein 3 | 81 |  |  | |
| NUCB2 | NUCB2 | Nucleobindin 2 | 98 |  |  | |
| ODC1 | ODC1 | Ornithine decarboxylase 1 | 75 |  |  | |
| PACSIN2 | PACSIN2 | Protein kinase C and casein kinase substrate in neurons 2 | 89 |  |  | |
| PCNT | PCNT | Pericentrin | 91 |  |  | |
| PDIA4 | PDIA4 | Protein disulfide-isomerase A4 | 290 |  |  | |
| PHB2 | PHB2 | Prohibitin 2 | 116 |  |  | |
| PIGR | PIGR | Polymeric immunoglobulin receptor | 159 |  |  | |
| PLCD1 | PLCD1 | Phospholipase C, delta 1 | 84 |  |  | |
| PLEC1 | PLEC1 | Plectin-1 | 78 |  |  | |
| PLG | PLG | Plasminogen | 76 |  |  | |
| PLS3 | PLS3 | Plastin 3 | 120 |  |  | |
| PPP2R3A | PPP2R3B | Protein phosphatase 2 (formerly 2A), regulatory subunit B | 85 |  |  | |
| PRKCDBP | PRKCDBP | Protein kinase C, delta | 100 |  |  | |
| PRP-2 | PRH1 | similar to Proline-rich protein | 134 |  |  | |
| PTGFRN | PTGFRN | Prostaglandin F2 receptor negative regulator | 115 |  |  | |
| PTPRC | PTPRC | Protein tyrosine phosphatase, receptor type, C | 83 |  |  | |
| PTPRJ | PTPRJ | Protein tyrosine phosphatase, receptor type, J | 76 |  |  | |
| QSER1 | QSER1 | Glutamine and serine-rich protein 1 | 91 |  |  | |
| RASIP1 | RASIP1 | Ras interacting protein 1 | 229 |  |  | |
| RGD1306866 | FAM38B | Family with sequence similarity 38, member B | 107 |  |  | |
| RGD1307525 | KIAA0408 | Hypothetical protein LOC9729 | 81 |  |  | |
| RGD1307736 | KIAA0152 | Hypothetical protein LOC9761 | 147 |  |  | |
| RGD1307772 | FAM65A | Family with sequence similarity 65, member A | 78 |  |  | |
| RGD1308168 | KIAA1618 | Hypothetical protein LOC57714 | 75 |  |  | |
| RGD1308274 | GVIN1 | GTPase, very large interferon inducible 1 | 88 |  |  | |
| RGD1310448 | STARD9 | StAR-related lipid transfer protein 9 | 76 |  |  | |
| RGD1311704 | C10ORF12 | Chromosome 10 open reading frame 12 | 90 |  |  | |
| RGD1560307 | LLGL2 | Lethal giant larvae homolog 2 | 108 |  |  | |
| RGD1561831 | TGM3 | Transglutaminase 3 | 120 |  |  | |
| RGD1562262 | CEP250 | Centrosomal protein 250kDa | 90 |  |  | |
| RGD1564216 | FER1L3 | Fer-1-like 3, myoferlin | 370 |  |  | |
| RGD1565368 | GAPDH | Glyceraldehyde-3-phosphate dehydrogenase | 154 |  |  | |
| RGD1566031 | PDZK10 | FERM and PDZ domain containing 4 | 85 |  |  | |
| ROCK1 | ROCK1 | Rho-associated, coiled-coil containing protein kinase 1 | 81 |  |  | |
| RRP1 | RRP1 | Ribosomal RNA processing 1 homolog | 76 |  |  | |
| RT1-CE15 | HLA-B | Major histocompatibility complex, class I, B | 79 |  |  | |
| RYR3 | RYR3 | Ryanodine receptor 3 | 89 |  |  | |
| SACM1L | SACM1L | Phosphatidylinositide phosphatase | 186 |  |  | |
| SDFR1 | NPTN | Neuroplastin (stromal cell derived factor receptor 1) | 106 |  |  | |
| SEC16A | SEC16A | SEC16 homolog A | 85 |  |  | |
| SEPT11 | SEPT11 | Septin 11 | 152 |  |  | |
| SEPT2 | SEPT2 | Septin 2 (NEDD5) | 78 |  |  | |
| SEPT4 | SEPT4 | Septin 4 | 88 |  |  | |
| SERPINA1 | SERPINA1 | Serpin peptidase inhibitor, clade A (alpha-1 antiproteinase, antitrypsin), member 1 | 100 |  |  | |
| SFTPD | SFTPD | Surfactant, pulmonary-associated protein D | 79 |  |  | |
| SLC25A3 | SLC25A3 | Solute carrier family 25 member 3 | 82 |  |  | |
| SLC9A3R1 | SLC9A3R1 | Solute carrier family 9 member 3 regulatory factor 1 | 97 |  |  | |
| SLCO2A1 | SLCO2A1 | Solute carrier organic anion transporter family, member 2A1 | 120 |  |  | |
| SLK | SLK | STE20-like kinase | 406 |  |  | |
| SORT1 | SORT1 | Sortilin 1 | 85 |  |  | |
| SPFH1 | SPFH1 | ER lipid raft associated 1 | 119 |  |  | |
| SPN | SPN | Sialophorin | 100 |  |  | |
| SPTBN2 | SPTBN2 | Spectrin, beta, non-erythrocytic 2 | 90 |  |  | |
| SRC | SRC | V-src sarcoma (Schmidt-Ruppin A-2) viral oncogene homolog | 103 |  |  | |
| SRF | PTK7 | Protein tyrosine kinase 7 | 89 |  |  | |
| STX12 | STX12 | Syntaxin-12 | 107 |  |  | |
| STX4A | STX4A | Syntaxin-binding protein 4 | 96 |  |  | |
| STX6 | STX6 | Syntaxin-6 | 75 |  |  | |
| STXBP2 | STXBP2 | Syntaxin binding protein 2 | 139 |  |  | |
| SYNE1 | SYNE1 | Spectrin repeat containing, nuclear envelope 1 | 90 |  |  | |
| SYNE2 | SYNE2 | Spectrin repeat containing, nuclear envelope 2 | 82 |  |  | |
| TDRD9 | TDRD9 | Tudor domain containing 9 | 107 |  |  | |
| TJP2 | TJP2 | Tight junction protein 2 | 75 |  |  | |
| TLOC1 | TLOC1 | SEC62 homolog | 79 |  |  | |
| TTC35 | TTC35 | Tetratricopeptide repeat protein 35 | 161 |  |  | |
| TTN | TTN | Titin | 146 |  |  | |
| TUBA1A | TUBA1A | Tubulin, alpha 1a | 378 |  |  | |
| TUBA4 | TUBA | Dynamin-binding protein | 75 |  |  | |
| TUBB2C | TUBB2C | Tubulin, beta 2C | 75 |  |  | |
| TUBB5 | TUBB5 | Tubulin beta-5 | 732 |  |  | |
| UBA52 | UBA52 | Ubiquitin A-52 residue ribosomal protein fusion product 1 | 90 |  |  | |
| UGCGL1 | UGCGL1 | UDP-glucose ceramide glucosyltransferase-like 1 | 202 |  |  | |
| UGT1A3 | UGT1A3 | UDP glucuronosyltransferase 1 family, polypeptide A6 | 167 |  |  | |
| UQCRC1 | UQCRC1 | Ubiquinol-cytochrome c reductase core protein I | 108 |  |  | |
| USP24 | USP24 | Ubiquitin carboxyl-terminal hydrolase 24 | 80 |  |  | |
| USP37 | USP37 | Ubiquitin specific peptidase 37 | 82 |  |  | |
| VNN1 | VNN1 | Vanin 1 | 140 |  |  | |
| YES1 | YES1 | Yamaguchi sarcoma viral oncogene homolog 1 | 124 |  |  | |
| ZAN | ZAN | zonadhesin | 78 |  |  | |
| ZFP91 | ZFP91 | Zinc finger protein 91 homolog | 95 |  |  | |
