# Supplementary material for: Comparative Proteomic Analysis of Lung Lamellar Bodies and Lysosome-Related Organelles
Source: PLoS One. 2011 Jan 26;6(1):e16482. doi: 10.1371/journal.pone.0016482 (PMC3027677; doi:10.1371/journal.pone.0016482)
Supplement: Table S2 — Subcellular localization of rat LB proteins selected from the gene ontology database (http://www.ebi.ac.uk/GOA/). (DOC) [file pone.0016482.s004.doc]

|  |  | Lamellar Body |  | |  | | |  |  | |
| --- | --- | --- | --- | --- | --- | --- | --- | --- | --- | --- |
|  |  |  | Mascot Score | | | | | | | |
| Rat Symbol | Human Symbol | NAME | LB | LME | | LMD | | | |  |
| ABCA3 | ABCA3 | ATP-binding cassette, sub-family A (ABC1), member 3 | 272 | 673 | | 120 | | | |  |
| LYZ | LYZ | Lysozyme | 199 | 98 | | 232 | | | |  |
| NAPSA | NAPSA | Napsin A aspartic peptidase | 101 |  | | 97 | | | |  |
| PRDX6 | PRDX6 | Peroxiredoxin-6 |  | 75 | | 469 | | | |  |
| SFTPA1 | SFTPA1 | Surfactant, pulmonary-associated protein A | 319 | 75 | | 466 | | | |  |
| SFTPB | SFTPB | Surfactant, pulmonary-associated protein B | 75 |  | | 156 | | | |  |
| SFTPC | SFTPC | Surfactant, pulmonary-associated protein C | 294 |  | |  | | | |  |
| SFTPD | SFTPD | Surfactant, pulmonary-associated protein D | 79 |  | |  | | | |  |
|  |  |  |  |  | |  | | | |  |
|  |  | Lysosome |  |  | |  | | | |  |
|  |  |  | Mascot Score | | | |  | | | |
| Rat Symbol | Human Symbol | NAME | LB | LME | | LMD | | | |  |
| AOC2 | AOC2 | Amine oxidase, copper containing 2 | 78 |  | | 213 | | | |  |
| AOC3 | AOC3 | Amine oxidase, copper containing 3 | 584 | 162 | |  | | | |  |
| ATP1A1 | ATP1A1 | ATPase, Na+/K+ transporting, alpha 1 | 348 | 672 | | 168 | | | |  |
| ATP6V0A1 | ATP6V0A1 | ATPase, H+ transporting, lysosomal V0 subunit a1 | 199 |  | |  | | | |  |
| ATP6V1B2 | ATP6V1B2 | ATPase, H+ transporting, lysosomal 56/58kDa, V1 subunit B2 | 77 |  | |  | | | |  |
| CALU | CALU | Calumenin |  | 75 | | 93 | | | |  |
| CANX | CANX | Calnexin | 431 | 438 | | 125 | | | |  |
| CAT | CAT | Catalase | 118 |  | |  | | | |  |
| CNP1 | CNP1 | 2',3'-cyclic nucleotide 3' phosphodiesterase | 162 |  | |  | | | |  |
| FASN | FASN | Fatty acid synthase | 103 |  | |  | | | |  |
| GANAB | GANAB | Neutral alpha-glucosidase AB precursor | 244 |  | | 88 | | | |  |
| GNB1 | GNB1 | Guanine nucleotide binding protein (G protein), beta polypeptide 1 | 328 | 346 | |  | | | |  |
| GNB2 | GNB2 | Guanine nucleotide binding protein (G protein), beta polypeptide 2 | 427 | 307 | |  | | | |  |
| GUSB | GUSB | Glucuronidase, beta |  | 75 | |  | | | |  |
| HSPCB | HSP90AB1 | Heat shock protein 90kDa alpha (cytosolic), class B member 1 |  | 75 | | 414 | | | |  |
| ITGA3 | ITGA3 | Integrin, alpha 3 |  | 94 | |  | | | |  |
| ITGB1 | ITGB1 | Integrin beta 1 | 544 | 264 | |  | | | |  |
| LAMP1 | LAMP1 | Lysosomal-associated membrane protein 1 | 135 |  | |  | | | |  |
| LOC362587 | MACF1 | Microtubule-actin crosslinking factor 1 | 86 |  | |  | | | |  |
| LOC500373 | CHMP4B | Chromatin modifying protein 4B | 154 |  | |  | | | |  |
| M6PR | M6PR | Mannose-6-phosphate receptor | 234 |  | |  | | | |  |
| METTL7A | METTL7A | Methyltransferase like 7A | 95 |  | |  | | | |  |
| MRC1 | MRC1 | Mannose receptor, C type 1 | 116 |  | |  | | | |  |
| P4HB | P4HB | Procollagen-proline, 2-oxoglutarate 4-dioxygenase (proline 4-hydroxylase), beta | 443 | 75 | | 384 | | | |  |
| PDIA4 | PDIA4 | Protein disulfide-isomerase A4 | 290 |  | |  | | | |  |
| RAC1 | RAC1 | Ras-related C3 botulinum toxin substrate 1 |  | 105 | |  | | | |  |
| RAP1A | RAP1A | Ras-related protein Rap-1A | 95 | 180 | | 86 | | | |  |
| RGD1306866 | FAM38B | Family with sequence similarity 38, member B | 107 |  | |  | | | |  |
| SCARB2 | SCARB2 | Scavenger receptor class B, member 2 | 243 |  | | 129 | | | |  |
| SORT1 | SORT1 | Sortilin 1 | 85 |  | |  | | | |  |
| SRC | SRC | V-src sarcoma (Schmidt-Ruppin A-2) viral oncogene homolog | 103 |  | |  | | | |  |
| SUSD2 | SUSD2 | Sushi domain-containing protein 2 | 223 | 189 | |  | | | |  |
| TF | TF | Transferrin | 664 | 153 | |  | | | |  |
| TKT | TKT | Transketolase | 253 | 75 | | 254 | | | |  |
| TRA1 | HSP90B1 | Heat shock protein 90kDa beta (Grp94), member 1 | 881 | 89 | | 416 | | | |  |
| YWHAE | YWHAE | Tyrosine 3-monooxygenase/tryptophan 5-monooxygenase activation protein, epsilon | 153 | 75 | | 197 | | | |  |
| YWHAH | YWHAH | Tyrosine 3-monooxygenase/tryptophan 5-monooxygenase activation protein, eta |  | 75 | |  | | | |  |
| YWHAQ | YWHAQ | Tyrosine 3-monooxygenase/tryptophan 5-monooxygenase activation protein, theta |  | 75 | |  | | | |  |
| YWHAZ | YWHAZ | Tyrosine 3-monooxygenase/tryptophan 5-monooxygenase activation protein, zeta |  | 75 | |  | | | |  |
|  |  |  |  |  | |  | | | |  |
|  |  | Vesicle |  |  | |  | | | |  |
|  |  |  | Mascot Score | | | | | | |  |
| Rat Symbol | Human Symbol | NAME | LB | LME | | LMD | | | |  |
| ACTR2 | ACTR2 | Actin-related protein 2 | 118 | 364 | |  | | | |  |
| ANXA1 | ANXA1 | Annexin A1 | 254 | 108 | | 200 | | | |  |
| ANXA11 | ANXA11 | Annexin A11 | 92 |  | |  | | | |  |
| ANXA2 | ANXA2 | Annexin A2 | 565 | 203 | | 338 | | | |  |
| ANXA3 | ANXA3 | Annexin A3 |  | 195 | | 163 | | | |  |
| ANXA4 | ANXA4 | Annexin A4 | 434 |  | |  | | | |  |
| ANXA5 | ANXA5 | Annexin A5 | 406 | 91 | | 671 | | | |  |
| ANXA6 | ANXA6 | Annexin A6 | 1551 | 496 | | 390 | | | |  |
| AP2A1 | AP2A1 | Adaptor-related protein complex 2, alpha 1 | 332 |  | |  | | | |  |
| AP2A2 | AP2A2 | Adaptor-related protein complex 2, alpha 2 | 589 |  | |  | | | |  |
| AP2B1 | AP2B1 | Adaptor-related protein complex 2, beta 1 | 356 | 86 | |  | | | |  |
| AP2M1 | AP2M1 | Adaptor-related protein complex 2, mu 1 | 116 |  | |  | | | |  |
| ATP2C2 | ATP2C2 | ATPase, Ca++ transporting, type 2C, member 2 |  | 108 | |  | | | |  |
| ATP8A1 | ATP8A1 | ATPase, aminophospholipid transporter (APLT), Class I, type 8A, member 1 | 163 | 119 | |  | | | |  |
| CALM2 | CALM2 | Calmodulin 2 |  | 75 | | 137 | | | |  |
| CDC42 | CDC42 | Cell division cycle 42 (GTP binding protein, 25kDa) | 104 | 131 | | 85 | | | |  |
| CLTC | CLTC | Clathrin, heavy chain | 1798 | 75 | | 630 | | | |  |
| CLTCL1 | CLTCL1 | Clathrin, heavy chain-like 1 | 360 | 75 | |  | | | |  |
| CR1 | CR1 | Complement component (3b/4b) receptor 1 |  | 91 | |  | | | |  |
| DNPEP | DNPEP | Aspartyl aminopeptidase |  | 75 | |  | | | |  |
| ECE1 | ECE1 | Endothelin converting enzyme 1 | 85 | 263 | |  | | | |  |
| EHD1 | EHD1 | EH-domain containing 1 | 447 | 221 | |  | | | |  |
| EHD2 | EHD2 | EH-domain containing 2 | 1371 | 447 | | 384 | | | |  |
| EHD4 | EHD4 | EH-domain containing 4 | 1019 |  | |  | | | |  |
| EXOC4 | EXOC4 | Exocyst complex component 4 | 82 |  | |  | | | |  |
| FLOT1 | FLOT1 | Flotillin 1 | 133 |  | |  | | | |  |
| FLOT2 | FLOT2 | Flotillin 2 | 243 |  | |  | | | |  |
| GDI2 | GDI2 | Rab GDP dissociation inhibitor beta | 87 |  | |  | | | |  |
| GNA13 | GNA13 | Guanine nucleotide binding protein (G protein), alpha 13 | 279 |  | |  | | | |  |
| GNA14 | GNA14 | Guanine nucleotide binding protein (G protein), alpha 14 | 259 |  | |  | | | |  |
| GNA15 | GNA15 | Guanine nucleotide binding protein (G protein), alpha 15 | 99 |  | |  | | | |  |
| GNAL | GNAL | Guanine nucleotide binding protein (G protein), alpha activating activity polypeptide | 125 |  | |  | | | |  |
| GNAS | GNAS | Guanine nucleotide-binding protein G(s) subunit alpha | 235 | 116 | |  | | | |  |
| GNB3 | GNB3 | Guanine nucleotide binding protein (G protein), beta polypeptide 3 | 191 |  | |  | | | |  |
| HSPA5 | HSPA5 | Heat shock 70kDa protein 5 | 1415 | 550 | | 774 | | | |  |
| HSPA8 | HSPA8 | Heat shock 70kDa protein 8 | 840 | 75 | | 274 | | | |  |
| KIF16B | C20ORF23 | Chromosome 20 open reading frame 23 | 93 |  | |  | | | |  |
| LMAN1L | LMAN1 | Lectin, mannose-binding, 1 | 245 |  | |  | | | |  |
| LMAN2L | LMAN2 | Lectin, mannose-binding, 2 | 133 |  | |  | | | |  |
| LRP1 | LRP1 | Low density lipoprotein-related protein 1 |  | 108 | |  | | | |  |
| LRP2 | LRP2 | Low density lipoprotein-related protein 2 | 94 | 78 | |  | | | |  |
| MARCKS | MARCKS | Myristoylated alanine-rich protein kinase C substrate | 402 | 75 | | 148 | | | |  |
| MBC2 | FAM62A | Family with sequence similarity 62 (C2 domain containing), member A | 557 | 77 | |  | | | |  |
| PACSIN2 | PACSIN2 | Protein kinase C and casein kinase substrate in neurons 2 | 89 |  | |  | | | |  |
| PTRF | PTRF | Polymerase I and transcript release factor |  | 75 | | 97 | | | |  |
| RAB1A | RAB1A | RAB1, member RAS oncogene family |  |  | | 115 | | | |  |
| RASIP1 | RASIP1 | Ras interacting protein 1 | 229 |  | |  | | | |  |
| RGD1308274 | GVIN1 | GTPase, very large interferon inducible 1 | 88 |  | |  | | | |  |
| SEC14L3 | SEC14L3 | SEC14-like 3 | 270 | 75 | | 136 | | | |  |
| SEC16A | SEC16A | SEC16 homolog A | 85 |  | |  | | | |  |
| SOD1 | SOD1 | Superoxide dismutase 1 |  |  | | 269 | | | |  |
| STX12 | STX12 | Syntaxin-12 | 107 |  | |  | | | |  |
| STX4A | STX4A | Syntaxin-binding protein 4 | 96 |  | |  | | | |  |
| STX6 | STX6 | Syntaxin-6 | 75 |  | |  | | | |  |
| STXBP2 | STXBP2 | Syntaxin binding protein 2 | 139 |  | |  | | | |  |
| TMED10 | TMED10 | Transmembrane emp24-like trafficking protein 10 | 98 |  | |  | | | |  |
| VAMP3 | VAMP3 | Vesicle-associated membrane protein 3 |  | 111 | |  | | | |  |
| VAT1 | VAT1 | Vesicle amine transport protein 1 homolog | 377 | 75 | |  | | | |  |
| VCP | VCP | Valosin-containing protein | 371 | 75 | | 233 | | | |  |
|  |  |  |  |  | |  | | | |  |
|  |  | Plasma Membrane |  |  | |  | | | |  |
|  |  |  | Mascot Score | | | | | | |  |
| Rat Symbol | Human Symbol | NAME | LB | LME | | LMD | | | |  |
| ABCA8A | ABCA8A | ATP-binding cassette sub-family A member 8-A |  | 107 | |  | | | |  |
| ACE | ACE | Angiotensin I converting enzyme | 1155 | 561 | | 233 | | | |  |
| ADAM10 | ADAM10 | ADAM metallopeptidase domain 10 | 77 |  | |  | | | |  |
| AGER | AGER | Advanced glycosylation end product-specific receptor | 179 | 123 | |  | | | |  |
| ALCAM | ALCAM | Activated leukocyte cell adhesion molecule | 161 |  | |  | | | |  |
| ALPL | ALPL | Alkaline phosphatase | 214 |  | |  | | | |  |
| APC | APC | Adenomatosis polyposis coli | 108 |  | |  | | | |  |
| APP | APP | Amyloid beta (A4) precursor protein | 92 |  | |  | | | |  |
| AQP1 | AQP1 | Aquaporin 1 |  |  | | 148 | | | |  |
| ATP1A3 | ATP1A3 | ATPase, Na+/K+ transporting, alpha 3 | 75 |  | |  | | | |  |
| ATP2A2 | ATP2A2 | ATPase, Ca++ transporting, cardiac muscle, slow twitch 2 | 118 | 214 | | 297 | | | |  |
| ATP2B1 | ATP2B1 | ATPase, Ca++ transporting, plasma membrane 1 | 304 | 233 | |  | | | |  |
| ATP2B2 | ATP2B2 | ATPase, Ca++ transporting, plasma membrane 2 | 109 |  | |  | | | |  |
| ATP2B4 | ATP2B4 | ATPase, Ca++ transporting, plasma membrane 4 | 364 | 163 | |  | | | |  |
| BCAM | BCAM | Basal cell adhesion molecule | 181 | 148 | |  | | | |  |
| CA4 | CA4 | Carbonic anhydrase IV | 206 |  | |  | | | |  |
| CABP1 | CABP1 | Calcium binding protein 1 | 371 |  | | 75 | | | |  |
| CAPN2 | CAPN2 | Calpain 2 |  |  | | 245 | | | |  |
| CAV | CAV1 | Caveolin 1 | 122 | 113 | | 116 | | | |  |
| CD31 | CD31 | Platelet/endothelial cell adhesion molecule (CD31 antigen) | 137 |  | | 105 | | | |  |
| CD36 | CD36 | CD36 molecule | 283 |  | |  | | | |  |
| COLEC12 | COLEC12 | Collectin-12 | 102 |  | |  | | | |  |
| CP | CP | Ceruloplasmin | 905 |  | | 136 | | | |  |
| CPM | CPM | Carboxypeptidase M | 139 |  | |  | | | |  |
| CTNNB1 | CTNNB1 | Beta-catenin | 202 |  | |  | | | |  |
| DPEP1 | DPEP1 | Dipeptidase 1 | 293 | 270 | |  | | | |  |
| DPP4 | DPP4 | Dipeptidyl-peptidase 4 | 1034 | 323 | |  | | | |  |
| ENO1 | ENO1 | Enolase 1 | 105 | 75 | |  | | | |  |
| ENPEP | ENPEP | Glutamyl aminopeptidase | 500 | 261 | |  | | | |  |
| ESAM | ESAM | Endothelial cell adhesion molecule |  | 101 | |  | | | |  |
| GNA11 | GNA11 | Guanine nucleotide-binding protein subunit alpha-11 | 166 | 166 | |  | | | |  |
| GNAI1 | GNAI1 | Guanine nucleotide binding protein, alpha inhibiting activity polypeptide 1 | 284 |  | |  | | | |  |
| GNAI2 | GNAI2 | Guanine nucleotide binding protein, alpha inhibiting activity polypeptide 2 | 783 |  | |  | | | |  |
| GNAI3 | GNAI3 | Guanine nucleotide binding protein, alpha inhibiting activity polypeptide 3 | 416 | 89 | |  | | | |  |
| GNAO1 | GNAO1 | Guanine nucleotide binding protein, alpha activating activity polypeptide O | 390 |  | |  | | | |  |
| GNAQ | GNAQ | Guanine nucleotide binding protein, q polypeptide | 318 | 187 | |  | | | |  |
| GNAT1 | GNAT1 | Guanine nucleotide binding protein, alpha transducing activity polypeptide 1 |  | 78 | |  | | | |  |
| GNAT2 | GNAT2 | Guanine nucleotide binding protein, alpha transducing activity polypeptide 2 | 203 |  | |  | | | |  |
| GNAT3 | GNAT3 | Guanine nucleotide binding protein, alpha transducing activity polypeptide 3 | 201 | 548 | |  | | | |  |
| GPIAP1 | GPIAP1 | GPI-anchored membrane protein 1 |  | 75 | |  | | | |  |
| GPRC5A | GPRC5A | G protein-coupled receptor, family C, group 5, member A | 204 |  | |  | | | |  |
| HDLBP | HDLBP | High density lipoprotein binding protein (vigilin) | 90 |  | |  | | | |  |
| ICAM1 | ICAM1 | Intercellular adhesion molecule 1 | 103 |  | |  | | | |  |
| ITGA1 | ITGA1 | Integrin alpha-1 | 252 |  | |  | | | |  |
| ITGAL | ITGAL | Integrin alpha-L | 105 |  | |  | | | |  |
| ITGAM | ITGAM | Integrin alpha-M | 138 |  | |  | | | |  |
| ITGAV | ITGAV | Integrin, alpha V |  | 100 | |  | | | |  |
| LNPEP | LNPEP | Leucyl/cystinyl aminopeptidase | 200 |  | |  | | | |  |
| LOC499912 | C20ORF3 | Chromosome 20 open reading frame 3 | 299 |  | |  | | | |  |
| MCAM | MCAM | Melanoma cell adhesion molecule | 96 |  | |  | | | |  |
| MME | MME | Membrane metallo-endopeptidase | 373 |  | |  | | | |  |
| PIGR | PIGR | Polymeric immunoglobulin receptor | 159 |  | |  | | | |  |
| PRKAR2A | PRKAR2A | Protein kinase, cAMP-dependent, regulatory, type II, alpha |  | 75 | |  | | | |  |
| PRX | PRX | Periaxin | 2184 | 746 | | 109 | | | |  |
| PTPRC | PTPRC | Protein tyrosine phosphatase, receptor type, C | 83 |  | |  | | | |  |
| PTPRJ | PTPRJ | Protein tyrosine phosphatase, receptor type, J | 76 |  | |  | | | |  |
| RGD1561831 | TGM3 | Transglutaminase 3 | 120 |  | |  | | | |  |
| RGD1564216 | FER1L3 | Fer-1-like 3, myoferlin | 370 |  | |  | | | |  |
| RT1-A2 | HLA-A | Major histocompatibility complex, class I, A | 285 | 87 | |  | | | |  |
| RT1-CE15 | HLA-B | Major histocompatibility complex, class I, B | 79 |  | |  | | | |  |
| RYR3 | RYR3 | Ryanodine receptor 3 | 89 |  | |  | | | |  |
| SDFR1 | NPTN | Neuroplastin (stromal cell derived factor receptor 1) | 106 |  | |  | | | |  |
| SDPR | SDPR | Serum deprivation-response protein | 575 | 75 | | 168 | | | |  |
| SLC3A2 | SLC3A2 | Solute carrier family 3 member 2 | 118 |  | |  | | | |  |
| SLC4A1 | SLC4A1 | Solute carrier family 4 member 1 | 393 |  | |  | | | |  |
| SLC6A14 | SLC6A14 | Solute carrier family 6 member 14 | 135 |  | | 75 | | | |  |
| SLC9A3R1 | SLC9A3R1 | Solute carrier family 9 member 3 regulatory factor 1 | 97 |  | |  | | | |  |
| SLCO2A1 | SLCO2A1 | Solute carrier organic anion transporter family, member 2A1 | 120 |  | |  | | | |  |
| SPN | SPN | Sialophorin | 100 |  | |  | | | |  |
| SRF | PTK7 | Protein tyrosine kinase 7 | 89 |  | |  | | | |  |
| THBD | THBD | Thrombomodulin | 211 | 163 | |  | | | |  |
| VNN1 | VNN1 | Vanin 1 | 140 |  | |  | | | |  |
| XPNPEP2 | XPNPEP2 | X-prolyl aminopeptidase (aminopeptidase P) 2, membrane-bound | 780 | 586 | |  | | | |  |
| YES1 | YES1 | Yamaguchi sarcoma viral oncogene homolog 1 | 124 |  | |  | | | |  |
|  |  |  |  |  | |  | | | |  |
|  |  | Secreted |  |  | |  | | | |  |
|  |  |  | Mascot Score | | | | | | |  |
| Rat Symbol | Human Symbol | NAME | LB | LME | | LMD | | | |  |
| A2M | A2M | Alpha-2-macroglobulin |  |  | | 99 | | | |  |
| ALB | ALB | Albumin | 1807 | 790 | | 337 | | | |  |
| APOE | APOE | Apolipoprotein E | 167 |  | |  | | | |  |
| APOH | APOH | Apolipoprotein H | 93 |  | |  | | | |  |
| C3 | C3 | Complement component 3 | 129 |  | | 178 | | | |  |
| CFB | CFB | Complement factor B | 75 |  | |  | | | |  |
| ENDOD1 | ENDOD1 | Endonuclease domain-containing 1 | 82 |  | |  | | | |  |
| FCRLS | FCRL2 | Fc receptor-like 2 |  | 156 | |  | | | |  |
| GDA | GDA | Guanine deaminase | 414 | 155 | | 272 | | | |  |
| HBA | HBA | Hemoglobin alpha-2 chain |  | 138 | |  | | | |  |
| HBB | HBB | Hemoglobin |  | 507 | | 661 | | | |  |
| ITGB2 | ITGB2 | Integrin beta-2 | 115 |  | |  | | | |  |
| LAMA5 | LAMA5 | Laminin, alpha 5 | 81 |  | |  | | | |  |
| LOC286911 | PRSS3 | Trypsin | 104 |  | |  | | | |  |
| PLG | PLG | Plasminogen | 76 |  | |  | | | |  |
| PON2 | PON2 | Paraoxonase 2 | 115 | 79 | |  | | | |  |
| PON3 | PON3 | Paraoxonase 3 | 300 | 127 | | 96 | | | |  |
| PRP-2 | PRH1 | similar to Proline-rich protein | 134 |  | |  | | | |  |
| PZP | PZP | Pregnancy-zone protein |  |  | | 153 | | | |  |
| RGD1564835 | HEPHL1 | Hephaestin-like 1 |  | 277 | |  | | | |  |
| SCPEP1 | SCPEP1 | Serine carboxypeptidase 1 |  |  | | 92 | | | |  |
| SERPINA1 | SERPINA1 | Serpin peptidase inhibitor, clade A (alpha-1 antiproteinase, antitrypsin), member 1 | 100 |  | |  | | | |  |
| SERPINA3N | SERPINA3 | Serpin peptidase inhibitor, clade A (alpha-1 antiproteinase, antitrypsin), member 3 | 130 |  | | 98 | | | |  |
| SERPINB6 | SERPINB6 | Serine (or cysteine) peptidase inhibitor, clade B, member 6a | 113 |  | | 91 | | | |  |
| SERPINH1 | SERPINH1 | Serpin peptidase inhibitor, clade H (heat shock protein 47), member 1, | 459 | 102 | |  | | | |  |
|  |  |  |  |  | |  | | | |  |
|  |  | Cytosol |  |  | |  | | | |  |
|  |  |  | Mascot Score | | | | | | |  |
| Rat Symbol | Human Symbol | NAME | LB | LME | | LMD | | | |  |
| ACACA | ACACA | Acetyl-CoA carboxylase 1 | 85 |  | |  | | | |  |
| AK1 | AK1 | Adenylate kinase 1 |  | 76 | |  | | | |  |
| AKAP5 | AKAP5 | A kinase (PRKA) anchor protein 5 | 136 |  | |  | | | |  |
| ALDH3A2 | ALDH3A2 | Aldehyde dehydrogenase 3 family, member A2 | 197 |  | |  | | | |  |
| ARHGEF5 | ARHGEF5 | Rho guanine nucleotide exchange factor (GEF) 5 | 81 |  | |  | | | |  |
| BLK | BLK | B lymphoid tyrosine kinase | 75 |  | |  | | | |  |
| BLVRB | BLVRB | Biliverdin reductase B |  |  | | 75 | | | |  |
| CA2 | CA2 | Carbonic anhydrase II |  |  | | 86 | | | |  |
| CDC37 | CDC37 | Cell division cycle 37 homolog | 108 |  | |  | | | |  |
| CHD9 | CHD9 | chromodomain helicase DNA binding protein 9 | 77 |  | |  | | | |  |
| CKB | CKB | Creatine kinase B-type | 111 |  | |  | | | |  |
| DDB1 | DDB1 | Damage-specific DNA binding protein 1, |  | 75 | |  | | | |  |
| DLC1 | DLC1 | Deleted in liver cancer 1 | 75 |  | |  | | | |  |
| DOCK6 | DOCK6 | Dedicator of cytokinesis protein 6 | 84 |  | |  | | | |  |
| EEF1A1 | EEF1A1 | Eukaryotic translation elongation factor 1 alpha 1 | 486 |  | |  | | | |  |
| EEF1A2 | EEF1A2 | Eukaryotic translation elongation factor 1 alpha 1 | 291 |  | |  | | | |  |
| EEF2 | EEF2 | Elongation factor 2 | 204 |  | |  | | | |  |
| EIF2C3 | EIF2C3 | Eukaryotic translation initiation factor 2C, 3 |  | 75 | |  | | | |  |
| EIF5A | EIF5A | Eukaryotic translation initiation factor 5A |  | 75 | |  | | | |  |
| FABP4 | FABP4 | Fatty acid binding protein 4 |  | 233 | | 258 | | | |  |
| FAM129A | FAM129A | Family with sequence similarity 129, member A, Niban | 200 |  | |  | | | |  |
| FKBP1A | FKBP1A | FK506 binding protein 1A |  | 75 | | 77 | | | |  |
| GCN1L1 | GCN1L1 | General control of amino-acid synthesis 1-like 1 | 82 |  | |  | | | |  |
| GSTA3 | GSTA3 | Glutathione S-transferase A3 |  | 75 | |  | | | |  |
| GSTP1 | GSTP1 | Glutathione S-transferase P |  |  | | 181 | | | |  |
| HERC1 | HERC1 | Guanine nucleotide exchange factor p532 | 84 |  | |  | | | |  |
| KCTD12 | KCTD12 | Potassium channel tetramerisation domain containing 12 | 103 | 75 | |  | | | |  |
| LDHA | LDHA | Lactate dehydrogenase A | 109 |  | | 125 | | | |  |
| LOC299949 | WDR67 | WD repeat domain 67 | 77 |  | |  | | | |  |
| LRRFIP2 | LRRFIP2 | Leucine rich repeat (in FLII) interacting protein 2 | 78 |  | |  | | | |  |
| LRRK1 | LRRK1 | Leucine-rich repeat kinase 1 | 95 |  | |  | | | |  |
| LYN | LYN | v-yes-1 Yamaguchi sarcoma viral related oncogene homolog | 449 | 176 | | 210 | | | |  |
| MDH1 | MDH1 | Malate dehydrogenase 1 | 78 |  | |  | | | |  |
| MVP | MVP | Major vault protein | 546 | 75 | | 456 | | | |  |
| NP | NP | Nnucleoside phosphorylase |  | 75 | | 225 | | | |  |
| ODC1 | ODC1 | Ornithine decarboxylase 1 | 75 |  | |  | | | |  |
| PHB2 | PHB2 | Prohibitin 2 | 116 |  | |  | | | |  |
| PLCD1 | PLCD1 | Phospholipase C, delta 1 | 84 |  | |  | | | |  |
| PLVAP | PLVAP | Plasmalemma vesicle associated protein | 285 |  | |  | | | |  |
| PPIA | PPIA | Peptidylprolyl isomerase A (cyclophilin A) |  | 221 | | 127 | | | |  |
| PPP1R14A | PPP1R14A | Protein phosphatase 1, regulatory (inhibitor) subunit 14A |  | 75 | |  | | | |  |
| Prdx2 | Prdx2 | Peroxiredoxin 2 |  | 75 | |  | | | |  |
| PSME3 | PSME3 | Proteasome (prosome, macropain) activator subunit 3 |  | 75 | |  | | | |  |
| PURA | PURA | Purine-rich element binding protein A |  | 75 | |  | | | |  |
| PURB | PURB | Purine-rich element binding protein B |  | 75 | |  | | | |  |
| RDX | RDX | Radixin | 707 |  | | 83 | | | |  |
| RGD1304704 | C14ORF166 | Chromosome 14 open reading frame 166 |  | 75 | |  | | | |  |
| RGD1307018 | FAM129B | Family with sequence similarity 129, member B, Niban-like |  | 93 | |  | | | |  |
| RGD1308168 | KIAA1618 | Hypothetical protein LOC57714 | 75 |  | |  | | | |  |
| RGD1311704 | C10ORF12 | Chromosome 10 open reading frame 12 | 90 |  | |  | | | |  |
| RGD1560307 | LLGL2 | Lethal giant larvae homolog 2 | 108 |  | |  | | | |  |
| RGD1562954 | AKR1C4 | Aldo-keto reductase family 1, member C4 | 85 |  | | 129 | | | |  |
| RHOA | RHOA | Ras homolog gene family, member A | 114 | 179 | |  | | | |  |
| RHOG | RHOG | Ras homolog gene family, member G |  | 244 | | 77 | | | |  |
| ROCK1 | ROCK1 | Rho-associated, coiled-coil containing protein kinase 1 | 81 |  | |  | | | |  |
| RRP1 | RRP1 | Ribosomal RNA processing 1 homolog | 76 |  | |  | | | |  |
| SEPT11 | SEPT11 | Septin 11 | 152 |  | |  | | | |  |
| SEPT2 | SEPT2 | Septin 2 (NEDD5) | 78 |  | |  | | | |  |
| SH3BGRL3 | SH3BGRL3 | SH3 domain binding glutamic acid-rich protein like 3 |  |  | | 82 | | | |  |
| SLK | SLK | STE20-like kinase | 406 |  | |  | | | |  |
| UBE1X | UBE1 | Ubiquitin-like modifier activating enzyme 1 | 125 |  | |  | | | |  |
| USP24 | USP24 | Ubiquitin carboxyl-terminal hydrolase 24 | 80 |  | |  | | | |  |
| USP37 | USP37 | Ubiquitin specific peptidase 37 | 82 |  | |  | | | |  |
| YBX1 | YBX1 | Y box binding protein 1 |  | 75 | |  | | | |  |
|  |  |  |  |  | |  | | | |  |
|  |  | Cytoskeleton |  |  | |  | | | |  |
|  |  |  | Mascot Score | | | | | | |  |
| Rat Symbol | Human Symbol | NAME | LB | LME | | LMD | | | |  |
| ACTA1 | ACTA1 | Actin, alpha 1, skeletal muscle | 662 |  | |  | | | |  |
| ACTB | ACTB | Beta-actin | 1252 | 640 | | 762 | | | |  |
| ACTG1 | ACTG1 | Actin, gamma 1 | 1102 |  | |  | | | |  |
| ACTN4 | ACTN4 | Actinin, alpha 4 |  | 463 | | 369 | | | |  |
| ANKRD7 | POTE2 | ANKRD26-like family C, member 1A | 480 |  | |  | | | |  |
| ARHGDIA | ARHGDIA | Rho GDP dissociation inhibitor (GDI) alpha |  | 75 | |  | | | |  |
| CAP350 | CAP350 | Centrosome-associated protein 350 | 97 |  | |  | | | |  |
| CATNA1 | CATNA1 | Alpha(E)-catenin | 433 |  | |  | | | |  |
| CD34 | CD34 | CD34 molecule | 153 | 82 | |  | | | |  |
| CD59 | CD59 | CD59 antigen |  | 81 | |  | | | |  |
| CD9 | CD9 | CD9 antigen | 75 | 91 | |  | | | |  |
| CDH13 | CDH13 | Cadherin 13 | 82 |  | |  | | | |  |
| CENPF | CENPF | Centromere protein F | 83 |  | |  | | | |  |
| CENTG1 | CENTG1 | Centaurin-gamma-1 | 75 |  | |  | | | |  |
| CFL1 | CFL1 | Cofilin 1 |  | 123 | |  | | | |  |
| CKAP4 | CKAP4 | Cytoskeleton-associated protein 4 | 694 | 93 | |  | | | |  |
| COL1A2 | COL1A2 | Collagen, type I, alpha 2, | 88 |  | |  | | | |  |
| COL6A3 | COL6A3 | Collagen, type VI, alpha 3 | 86 |  | |  | | | |  |
| COL9A2 | COL9A2 | Collagen, type IX, alpha 2, | 78 |  | |  | | | |  |
| CYFIP1 | CYFIP1 | Cytoplasmic FMR1 interacting protein 1 | 250 |  | |  | | | |  |
| CYLD | CYLD | Cylindromatosis | 125 |  | |  | | | |  |
| DCHS1 | DCHS1 | Protocadherin-16 | 119 |  | |  | | | |  |
| DMD | DMD | Dystrophin | 94 |  | |  | | | |  |
| DNAH1 | DNAH1 | Dynein, axonemal, heavy chain 1 | 89 |  | |  | | | |  |
| DNAH10 | DNAH10 | Dynein, Axonemal, heavy chain 10 | 89 |  | |  | | | |  |
| DPYSL2 | DPYSL2 | Dihydropyrimidinase-like 2 | 409 |  | | 263 | | | |  |
| DST | DST | Dystonin | 124 |  | |  | | | |  |
| DSTN | DSTN | Destrin |  | 75 | |  | | | |  |
| EZR | EZR | Ezrin | 80 | 75 | | 150 | | | |  |
| FLNA | FLNA | Filamin A, alpha |  | 75 | |  | | | |  |
| GSN | GSN | Gelsolin | 347 | 75 | | 183 | | | |  |
| HSPB1 | HSPB1 | Heat shock protein beta-1 |  | 75 | |  | | | |  |
| IQGAP1 | IQGAP1 | IQ motif containing GTPase activating protein 1 | 1297 | 125 | | 150 | | | |  |
| JUP | JUP | Junction plakoglobin | 160 |  | |  | | | |  |
| KB1 | KRT1 | Keratin | 118 | 75 | |  | | | |  |
| KIF15 | KIF15 | Kinesin family member 15 | 79 |  | |  | | | |  |
| KIF26A | KIF26A | Kinesin family member 26A | 83 |  | |  | | | |  |
| KTN1 | KTN1 | Kinectin 1 | 85 |  | |  | | | |  |
| LCP1 | LCP1 | Lymphocyte cytosolic protein 1 (L-plastin) | 87 | 75 | | 200 | | | |  |
| LMO7 | LMO7 | LIM domain 7 |  | 75 | |  | | | |  |
| LOC500363 | TUBA2 | Tubulin, alpha 3d | 203 |  | |  | | | |  |
| MGC109519 | TPM2 | Tropomyosin 2 |  | 75 | | 148 | | | |  |
| MRLCB | MRCL3 | Myosin regulatory light chain MRCL3 | 75 |  | |  | | | |  |
| MSN | MSN | Moesin | 1967 | 75 | | 577 | | | |  |
| MYH10 | MYH10 | Myosin, heavy chain 10 | 447 | 250 | |  | | | |  |
| MYH11 | MYH11 | Myosin, heavy chain 11 | 2411 | 114 | | 744 | | | |  |
| MYH14 | MYH14 | Myosin, heavy chain 14 | 1651 |  | |  | | | |  |
| MYH2 | MYH2 | Myosin, heavy chain 2 | 75 |  | |  | | | |  |
| MYH9 | MYH9 | Myosin, heavy polypeptide 9, non-muscle |  | 94 | |  | | | |  |
| MYL6 | MYL6 | Myosin, light chain 6, alkali, smooth muscle and non-muscle |  | 117 | |  | | | |  |
| MYL9 | MYL9 | Myosin, light chain 9, regulatory |  | 84 | |  | | | |  |
| MYO1B | MYO1B | Myosin IB |  | 97 | |  | | | |  |
| MYO1C | MYO1C | Myosin IC | 1247 |  | |  | | | |  |
| MYO6 | MYO6 | Myosin VI | 246 |  | |  | | | |  |
| NEB | NEB | Nebulin | 96 |  | |  | | | |  |
| PCNT | PCNT | Pericentrin | 91 |  | |  | | | |  |
| PFN1 | PFN1 | Profilin 1 |  | 75 | |  | | | |  |
| PLEC1 | PLEC1 | Plectin-1 | 78 |  | |  | | | |  |
| PLS3 | PLS3 | Plastin 3 | 120 |  | |  | | | |  |
| RGD1310121 | TPPP | Tubulin polymerization promoting protein |  | 75 | |  | | | |  |
| RGD1562262 | CEP250 | Centrosomal protein 250kDa | 90 |  | |  | | | |  |
| RGD1563977 | EPB41L2 | Erythrocyte membrane protein band 4.1-like 2 | 648 | 171 | |  | | | |  |
| RGD1566031 | PDZK10 | FERM and PDZ domain containing 4 | 85 |  | |  | | | |  |
| S100A11 | S100A11 | S100 calcium binding protein A11 | 75 |  | |  | | | |  |
| SEPT4 | SEPT4 | Septin 4 | 88 |  | |  | | | |  |
| SEPT7 | SEPT7 | Septin 7 | 292 |  | |  | | | |  |
| SPNA2 | SPTAN1 | Spectrin, alpha, non-erythrocytic 1 |  |  | | 98 | | | |  |
| SPTAN1 | SPTA1 | Spectrin, alpha, erythrocytic 1 |  | 75 | |  | | | |  |
| SPTBN2 | SPTBN2 | Spectrin, beta, non-erythrocytic 2 | 90 |  | |  | | | |  |
| SPTBN5 | SPTBN5 | Spectrin, beta, non-erythrocytic 5 | 110 | 75 | |  | | | |  |
| SYNE1 | SYNE1 | Spectrin repeat containing, nuclear envelope 1 | 90 |  | |  | | | |  |
| SYNE2 | SYNE2 | Spectrin repeat containing, nuclear envelope 2 | 82 |  | |  | | | |  |
| TAGLN2 | TAGLN2 | Transgelin-2 |  | 75 | |  | | | |  |
| TJP2 | TJP2 | Tight junction protein 2 | 75 |  | |  | | | |  |
| TLN1 | TLN1 | Talin 1 | 471 | 458 | |  | | | |  |
| TPM1 | TPM1 | Tropomyosin 1 |  | 75 | | 164 | | | |  |
| TTN | TTN | Titin | 146 |  | |  | | | |  |
| TUBA1A | TUBA1A | Tubulin, alpha 1a | 378 |  | |  | | | |  |
| TUBA4 | TUBA | Dynamin-binding protein | 75 |  | |  | | | |  |
| TUBB2C | TUBB2C | Tubulin, beta 2C | 75 |  | |  | | | |  |
| TUBB5 | TUBB5 | Tubulin beta-5 | 732 |  | |  | | | |  |
| VCL | VCL | Vinculin | 418 | 75 | | 166 | | | |  |
| ZAN | ZAN | zonadhesin | 78 |  | |  | | | |  |
| ZYX | ZYX | Zyxin |  | 75 | |  | | | |  |
|  |  |  |  |  | |  | | | |  |
|  |  | Endoplasmic Reticulum |  |  | |  | | | |  |
|  |  |  | Mascot Score | | | | | | |  |
| Rat Symbol | Human Symbol | NAME | LB | LME | | LMD | | | |  |
| ANPEP | ANPEP | Alanyl (membrane) aminopeptidase | 956 | 956 | |  | | | |  |
| ARTS1 | ARTS1 | Type 1 tumor necrosis factor receptor shedding aminopeptidase regulator | 121 |  | |  | | | |  |
| AYTL2 | AYTL2 | Acyltransferase like 2 | 107 |  | |  | | | |  |
| CALR | CALR | Calreticulin | 212 |  | |  | | | |  |
| CDIPT | CDIPT | CDP-diacylglycerol--inositol 3-phosphatidyltransferase | 93 |  | |  | | | |  |
| CES3 | CES3 | Carboxylesterase 3 | 556 |  | |  | | | |  |
| CYB5B | CYB5B | cytochrome b5 type B |  | 138 | | 157 | | | |  |
| CYB5R2 | CYB5R2 | NADH-cytochrome b5 reductase 2 |  | 75 | |  | | | |  |
| CYB5R3 | CYB5R3 | Cytochrome b5 reductase 3 | 291 | 75 | |  | | | |  |
| CYP2A3A | CYP2A13 | Cytochrome P450, family 2, subfamily A, polypeptide 13 | 75 |  | |  | | | |  |
| CYP2B6 | CYP2B6 | Cytochrome P450, family 2, subfamily B, polypeptide 6 |  | 500 | |  | | | |  |
| CYP2D6 | CYP2D6 | Cytochrome P450, family 2, subfamily D, polypeptide 6 |  | 86 | |  | | | |  |
| CYP2F2 | CYP2F2 | Cytochrome P450, family 2, subfamily f, polypeptide 2 | 252 |  | |  | | | |  |
| CYP2F4 | CYP2F1 | Cytochrome P450, family 2, subfamily f, polypeptide 4 | 342 |  | |  | | | |  |
| CYP2S1 | CYP2S1 | Cytochrome P450, family 2, subfamily S, polypeptide 1 | 139 |  | |  | | | |  |
| CYP4B1 | CYP4B1 | Cytochrome P450, family 4, subfamily B, polypeptide 1 | 174 | 75 | | 177 | | | |  |
| DDOST | DDOST | Dolichyl-diphosphooligosaccharide-protein glycosyltransferase | 279 |  | |  | | | |  |
| EFHD2 | EFHD2 | EF-hand domain family, member D2 |  | 75 | |  | | | |  |
| EPHX1 | EPHX1 | Epoxide hydrolase 1 | 301 |  | |  | | | |  |
| ERLIN1 | ERLIN1 | ER lipid raft associated 1 | 104 |  | |  | | | |  |
| ERLIN2 | ERLIN2 | ER lipid raft associated 2 | 194 |  | |  | | | |  |
| FMO1 | FMO1 | Flavin containing monooxygenase 1 | 268 |  | |  | | | |  |
| GCS1 | GCS1 | Glucosidase I | 213 |  | |  | | | |  |
| HSD11B1 | HSD11B1 | Hydroxysteroid (11-beta) dehydrogenase 1 | 191 |  | |  | | | |  |
| HSPA1A | HSPA1A | Heat shock 70kDa protein 1A | 288 |  | |  | | | |  |
| HSPA1B | HSPA6 | Heat shock 70kDa protein 6 | 278 |  | |  | | | |  |
| HSPCA | HSP90AA1 | Heat shock protein 90kDa alpha (cytosolic), class A member 1 |  | 75 | | 238 | | | |  |
| HYOU1 | HYOU1 | Hypoxia up-regulated protein 1 | 214 |  | | 98 | | | |  |
| MGC94145 | C1ORF179 | Chromosome 1 open reading frame 179 | 166 |  | |  | | | |  |
| MGST1 | MGST1 | Microsomal glutathione S-transferase 1 |  | 115 | |  | | | |  |
| NUCB1 | NUCB1 | Nucleobindin 1 | 141 |  | | 89 | | | |  |
| NUCB2 | NUCB2 | Nucleobindin 2 | 98 |  | |  | | | |  |
| PDIA3 | PDIA3 | Protein disulfide isomerase family A, member 3 | 882 | 114 | | 434 | | | |  |
| PDIA6 | PDIA6 | Protein disulfide isomerase associated 6 |  | 105 | |  | | | |  |
| PGRMC1 | PGRMC1 | Progesterone receptor membrane component 1 |  | 179 | |  | | | |  |
| POR | POR | P450 (cytochrome) oxidoreductase | 494 |  | | 111 | | | |  |
| PRKCSH | PRKCSH | Protein kinase C substrate 80K-H | 86 |  | | 75 | | | |  |
| PTGFRN | PTGFRN | Prostaglandin F2 receptor negative regulator | 115 |  | |  | | | |  |
| PTGIS | PTGIS | Prostacyclin synthase |  | 77 | |  | | | |  |
| RCN1 | RCN1 | Reticulocalbin-1 |  | 75 | |  | | | |  |
| RCN2 | RCN2 | Reticulocalbin-2 |  | 75 | | 118 | | | |  |
| RCN3 | RCN3 | Reticulocalbin-3 |  | 75 | |  | | | |  |
| RGD1307525 | KIAA0408 | Hypothetical protein LOC9729 | 81 |  | |  | | | |  |
| RGD1307736 | KIAA0152 | Hypothetical protein LOC9761 | 147 |  | |  | | | |  |
| RPN1 | RPN1 | Ribophorin I | 280 | 130 | |  | | | |  |
| RPN2 | RPN2 | Ribophorin II | 234 |  | | 209 | | | |  |
| RRBP1 | RRBP1 | Ribosome binding protein 1 | 151 |  | |  | | | |  |
| RTN4 | RTN4 | Reticulon 4 | 183 |  | |  | | | |  |
| SACM1L | SACM1L | Phosphatidylinositide phosphatase | 186 |  | |  | | | |  |
| SDF2L1 | SDF2L1 | Stromal cell-derived factor 2-like 1 |  | 127 | |  | | | |  |
| SPFH1 | SPFH1 | ER lipid raft associated 1 | 119 |  | |  | | | |  |
| TLOC1 | TLOC1 | SEC62 homolog | 79 |  | |  | | | |  |
| TMEM43 | TMEM43 | Transmembrane protein 43 | 137 |  | |  | | | |  |
| TTC35 | TTC35 | Tetratricopeptide repeat protein 35 | 161 |  | |  | | | |  |
| TXNDC4 | TXNDC4 | Thioredoxin domain-containing protein 4 | 139 |  | | 254 | | | |  |
| UBA52 | UBA52 | Ubiquitin A-52 residue ribosomal protein fusion product 1 | 90 |  | |  | | | |  |
| UGCGL1 | UGCGL1 | UDP-glucose ceramide glucosyltransferase-like 1 | 202 |  | |  | | | |  |
| UGT1A3 | UGT1A3 | UDP glucuronosyltransferase 1 family, polypeptide A6 | 167 |  | |  | | | |  |
|  |  |  |  |  | |  | | | |  |
|  |  | Unknown |  |  | |  | | | |  |
|  |  |  | Mascot Score | | | | | | |  |
| Rat Symbol | Human Symbol | NAME | LB | LME | | LMD | | | |  |
| ALDH3B1 | ALDH3B1 | Aldehyde dehydrogenase 3 family, member B1 | 158 | 164 | |  | | | |  |
| ALDOAL1 | ALDOA | Aldolase A, fructose-bisphosphate | 263 | 124 | |  | | | |  |
| ALDOC | ALDOC | Aldolase C, fructose-bisphosphate | 175 |  | |  | | | |  |
| ARHGAP23 | ARHGAP23 | Rho GTPase activating protein 23 | 85 |  | |  | | | |  |
| CRIP2 | CRIP2 | Cysteine-rich protein 2 |  | 75 | |  | | | |  |
| IGSF4D | IGSF4D | Cell adhesion molecule 2 |  | 90 | |  | | | |  |
| LOC287867 | CCDC40 | Coiled-coil domain containing 40 | 75 |  | |  | | | |  |
| MAP3K1 | MAP3K1 | Mitogen-activated protein kinase kinase kinase 1 | 82 |  | |  | | | |  |
| MGC72560 | C11ORF59 | Chromosome 11 open reading frame 59 |  | 78 | |  | | | |  |
| NRIP3 | NRIP3 | Nuclear receptor interacting protein 3 | 81 |  | |  | | | |  |
| PRKCDBP | PRKCDBP | Protein kinase C, delta | 100 |  | |  | | | |  |
| QSER1 | QSER1 | Glutamine and serine-rich protein 1 | 91 |  | |  | | | |  |
| RGD1307772 | FAM65A | Family with sequence similarity 65, member A | 78 |  | |  | | | |  |
| RGD1309871 | ATL3 | Atlastin 3 |  | 96 | |  | | | |  |
| RGD1310448 | STARD9 | StAR-related lipid transfer protein 9 | 76 |  | |  | | | |  |
| RGD1311703 | C11ORF58 | Chromosome 11 open reading frame 58 |  | 75 | |  | | | |  |
| RSAFD1 | LOC650433 | Similar to Radical S-adenosyl methionine and flavodoxin domains 1 |  | 282 | |  | | | |  |
| TDRD9 | TDRD9 | Tudor domain containing 9 | 107 |  | |  | | | |  |
|  |  |  |  |  | |  | | | |  |
|  |  | Nucleus |  |  | |  | | | |  |
|  |  |  | Mascot Score | | | | | | |  |
| Rat Symbol | Human Symbol | NAME | LB | LME | | LMD | | | |  |
| BRCA1 | BRCA1 | Breast cancer 1, early onset | 109 |  | |  | | | |  |
| FMR1 | FMR1 | Fragile X mental retardation 1 protein |  | 75 | |  | | | |  |
| GTF2H4 | GTF2H4 | General transcription factor IIH, polypeptide 4 |  | 75 | |  | | | |  |
| HNRNPA2B1 | HNRNPA2B1 | Heterogeneous nuclear ribonucleoprotein A2/B1 | 76 | 75 | | 95 | | | |  |
| HNRNPR | HNRNPR | Heterogeneous nuclear ribonucleoprotein R |  | 75 | |  | | | |  |
| HNRPA3 | HNRPA3 | Heterogeneous nuclear ribonucleoprotein A3 |  | 75 | |  | | | |  |
| LOC363267 | ZFP106 | Zinc finger protein 106 homolog | 80 |  | |  | | | |  |
| LOC686653 | ZNF596 | Zinc finger protein 596 | 92 |  | |  | | | |  |
| LOC691499 | ZNF469 | Zinc finger protein 469 | 79 |  | |  | | | |  |
| NCOR1 | NCOR1 | Nuclear receptor co-repressor 1 | 76 |  | |  | | | |  |
| PPP2R3A | PPP2R3B | Protein phosphatase 2 (formerly 2A), regulatory subunit B | 85 |  | |  | | | |  |
| RGD1565368 | GAPDH | Glyceraldehyde-3-phosphate dehydrogenase | 154 |  | |  | | | |  |
| ZFP91 | ZFP91 | Zinc finger protein 91 homolog | 95 |  | |  | | | |  |
|  |  |  |  |  | |  | | | |  |
|  |  | Mitochondrion |  |  | |  | | | |  |
|  |  |  | Mascot Score | | | | | | |  |
| Rat Symbol | Human Symbol | NAME | LB | LME | | LMD | | | |  |
| AKAP1 | AKAP1 | A kinase (PRKA) anchor protein 1 |  | 208 | |  | | | |  |
| ATP5A1 | ATP5A1 | ATP synthase, H+ transporting, mitochondrial F1 complex, alpha subunit 1, | 197 | 105 | |  | | | |  |
| ATP5B | ATP5B | ATP synthase, H+ transporting, mitochondrial F1 complex, beta | 242 |  | | 146 | | | |  |
| ATP5J | ATP5J | ATP synthase, H+ transporting, mitochondrial F0 complex, subunit F6 |  | 75 | |  | | | |  |
| IMMT | IMMT | Mitochondrial inner membrane protein | 85 |  | |  | | | |  |
| NDUFS1 | NDUFS1 | NADH dehydrogenase (ubiquinone) Fe-S protein 1 | 85 |  | |  | | | |  |
| NDUFV1 | NDUFV1 | NADH dehydrogenase (ubiquinone) flavoprotein 1 |  | 75 | |  | | | |  |
| NNT | NNT | Nicotinamide nucleotide transhydrogenase | 76 |  | |  | | | |  |
| PHB | PHB | Prohibitin |  | 89 | |  | | | |  |
| SLC25A3 | SLC25A3 | Solute carrier family 25 member 3 | 82 |  | |  | | | |  |
| UQCRC1 | UQCRC1 | Ubiquinol-cytochrome c reductase core protein I | 108 |  | |  | | | |  |
|  |  |  |  |  | |  | | | |  |
|  |  | Golgi |  |  | |  | | | |  |
|  |  |  | Mascot Score | | | | | | |  |
| Rat Symbol | Human Symbol | NAME | LB | LME | | LMD | | | |  |
| GLG1 | GLG1 | Golgi apparatus protein 1 | 160 |  | |  | | | |  |
| LOC680692 | GOLM1 | Golgi membrane protein 1 | 279 |  | |  | | | |  |
|  |  |  |  |  | |  | | | |  |
|  |  |  |  |  | |  | | | |  |
|  |  | Ribosome |  |  | |  | | | |  |
|  |  |  | Mascot Score | | | | | | |  |
| Rat Symbol | Human Symbol | NAME | LB | LME | | LMD | | | |  |
| RPL14 | RPL14 | Ribosomal protein L14 |  | 209 | |  | | | |  |
| RPL21 | RPL21 | Ribosomal protein L21 | 81 | 143 | |  | | | |  |
| RPL3 | RPL3 | Ribosomal protein L3 | 90 |  | |  | | | |  |
| RPL5 | RPL5 | Ribosomal protein L5 | 103 | 80 | |  | | | |  |
| RPL6 | RPL6 | Ribosomal protein L6 |  | 94 | |  | | | |  |
| RPLP1 | RPLP1 | Ribosomal protein, large, P1 |  | 81 | |  | | | |  |
| RPLP2 | RPLP2 | Ribosomal protein, large P2 |  | 131 | |  | | | |  |
| RPS10 | RPS10 | Ribosomal protein S10 |  |  | | 160 | | | |  |
| RPS15 | RPS15 | Ribosomal protein S15 |  | 194 | |  | | | |  |
| Rps19 | Rps19 | Ribosomal protein S19 |  | 150 | |  | | | |  |
| RPS2 | RPS2 | Ribosomal protein S2 | 111 |  | |  | | | |  |
| RPS3 | RPS3 | Ribosomal protein S3 | 98 |  | |  | | | |  |
| RPS3A | RPS3a | Ribosomal protein S3a | 226 |  | |  | | | |  |
