# Supplementary material for: Comparative Proteomic Analysis of Lung Lamellar Bodies and Lysosome-Related Organelles
Source: PLoS One. 2011 Jan 26;6(1):e16482. doi: 10.1371/journal.pone.0016482 (PMC3027677; doi:10.1371/journal.pone.0016482)
Supplement: Table S3 — LB proteins detected only by PCT extraction of the LB sample. (DOC) [file pone.0016482.s005.doc]

|  |  |  | Mascot Score |
| --- | --- | --- | --- |
| Rat Symbol | Human Symbol | NAME | LB |
| Aldh2 | Aldh2 | Aldehyde dehydrogenase 2, mitochondrial | 329 |
| Arf1 | Arf1 | ADP-ribosylation factor 1, isoform CRA_d | 359 |
| ARF4 | Arf4 | Unnamed protein product | 356 |
| Arg1 | Arg1 | Arginase 1, liver | 159 |
| ARL8B | ARL8B | ADP-ribosylation factor-like 10C | 257 |
| Asl | Asl | Argininosuccinate lyase | 229 |
| Atp5o | Atp5o | ATP synthase, H+ transporting, mitochondrial F1 complex, O subunit | 141 |
| Atp6v1e1 | Atp6v1e1 | Vacuolar H+ ATPase E1 | 133 |
| Bhmt | Bhmt | Betaine-homocysteine methyltransferase | 130 |
| Ca3 | Ca3 | Carbonic anhydrase 3 | 685 |
| Calm3 | Calm3 | Troponin C-like protein | 404 |
| Cap1 | Cap1 | CAP, adenylate cyclase-associated protein 1 | 166 |
| CD38 | CD38 | CD38 antigen | 106 |
| CES1 | LOC291863 | Carboxylesterase-like | 699 |
| CGN | CGN | Cingulin (predicted) | 107 |
| CLIC1 | CLIC1 | Chloride intracellular channel 1 | 98 |
| CLIC4 | CLIC4 | Chloride intracellular channel 4 | 150 |
| Cox4i1 | Cox4i1 | Cytochrome c oxidase subunit IV isoform 1 | 112 |
| Cps1 | Cps1 | Carbamoyl-phosphate synthetase 1 | 863 |
| Cr1l | Cr1l | Complement receptor related protein isoform 2 | 128 |
| Csad | Csad | Cysteine sulfinic acid decarboxylase | 190 |
| Ctnna1 | Ctnna1 | Catenin (cadherin-associated protein), alpha 1, 102kDa | 676 |
| Ctsb | Ctsb | Cathepsin B preproprotein | 122 |
| Ctsc | Ctsc | Cathepsin C | 125 |
| Cyp2b1 | Cyp2b1 | Cytochrome p-450 | 787 |
| ERGIC1 | LOC682838 | rCG34297 | 109 |
| Etfa | Etfa | Electron transferring flavoprotein, alpha polypeptide | 102 |
| Faah | Faah | Fatty acid amide hydrolase | 110 |
| Fabp1 | Fabp1 | Fatty acid binding protein 1, liver | 840 |
| Fbp1 | Fbp1 | Fructose bisphosphatase 1 | 123 |
| Fmo2 | Fmo2 | Flavin-containing monooxygenase 2 | 267 |
| Fmo3 | Fmo3 | Flavin containing monooxygenase 3 | 138 |
| Ftl1 | Ftl1 | Ferritin light chain 1 | 131 |
| Gc | Gc | Group specific component | 107 |
| Gm2a | Gm2a | GM2 ganglioside activator protein | 117 |
| Gstm1 | Gstm1 | Glutathione S-transferase, mu 1 | 565 |
| H2-K1 | H2-K1 | MHC H-2K antigen | 108 |
| H3F3A | H3F3A | H3 histone, family 3A | 160 |
| HAAO | Haao | 3-hydroxyanthranilate 3,4-dioxygenase | 131 |
| Hadh | Hadh | Hadh protein | 120 |
| HIST1H3A | Hist1h2ail | PREDICTED: similar to histone 1, H2ai (predicted) isoform 1 | 260 |
| HIST1H4A | Hist1h4b | PREDICTED: similar to germinal histone H4 gene | 155 |
| HIST2H2AC | HIST2H2AC | Histone cluster 2, H2ac | 241 |
| Hla-dmb | Hla-dmb | Major histocompatibility complex, class II, DM beta | 114 |
| HRSP12 | Hrsp12 | Heat-responsive protein | 181 |
| Hsd17b10 | Hsd17b10 | Amyloid beta-peptide binding protein | 111 |
| Ifitm3 | Ifitm3 | Interferon-inducible protein variant 10 | 165 |
| Kras | Kras | PREDICTED: similar to turkey K-Ras | 421 |
| Lyz2 | Lyz2 | Lysozyme C type 1 precursor (1,4-beta-N-acetylmuramidase C) | 764 |
| MDH2 | MDH2 | Malate dehydrogenase, mitochondrial precursor | 131 |
| Mfap4 | Mfap4 | Microfibrillar-associated protein 4 | 173 |
| MRCL2 | 2900073G15Rik | Myosin light chain, regulatory B-like | 763 |
| Myadm | Myadm | Myeloid-associated differentiation marker | 188 |
| Myh6 | Myh6 | Myosin-6 (Myosin heavy chain 6) (Myosin heavy chain, cardiac muscle alpha isoform) (MyHC-alpha) | 161 |
| Myo1d | Myo1d | Myosin ID | 454 |
| Myo1e | Myo1e | Myosin IE | 151 |
| Nup62cl | Nup62cl | Nucleoporin 62 C-terminal like | 95 |
| Pcyox1 | Pcyox1 | Prenylcysteine oxidase 1 | 100 |
| PDIA5 | PDIA5 | Protein disulfide isomerase-related protein 5 | 306 |
| Pgrmc2 | Pgrmc2 | Progesterone receptor membrane component 2 | 142 |
| Plp2 | Plp2 | Proteolipid protein 2 | 115 |
| Ppib | Ppib | Peptidylprolyl isomerase B | 499 |
| Ppp2r1a | Ppp2r1a | Unnamed protein product | 235 |
| Psma2 | Psma2 | Proteasome (prosome, macropain) subunit, alpha type 2 | 104 |
| Ptgis | Ptgis | Prostaglandin I2 synthase | 124 |
| Pygl | Pygl | Liver glycogen phosphorylase | 164 |
| RAB10 | RAB10 | RAB10, member RAS oncogene family | 199 |
| Rab11a | Rab11a | Small GTPase | 222 |
| RAB11B | Rab11b | RAB11B, member RAS oncogene family | 382 |
| Rab14 | Rab14 | RAB14, member RAS oncogene family | 726 |
| RAB18 | RAB18 | RAB18, member RAS oncogene family | 440 |
| RAB21 | RAB21 | RAB21, member RAS oncogene family | 132 |
| RAB2A | LOC100029760 | PREDICTED: hypothetical protein | 461 |
| RAB35 | RAB35 | RAB35, member RAS oncogene family | 166 |
| RAB5B | RAB5B | RAB5B, member RAS oncogene family | 159 |
| RAB5C | RAB5C | RAB5C, member RAS oncogene family | 207 |
| RAB7 | RAB7 | RAB7, member RAS oncogene family | 453 |
| Ralb | Ralb | V-ral simian leukemia viral oncogene homolog B (ras related) | 135 |
| Rap1b | Rap1b | RAS related protein 1b | 364 |
| RT1-Aw2 | RT1-Aw2 | MHC class Ia protein | 140 |
| Sec22b | Sec22b | SEC22 vesicle trafficking protein-like 1 | 231 |
| SLC25A4 | SLC25A4 | Solute carrier family 25 (mitochondrial carrier; adenine nucleotide translocator), member 4 | 148 |
| Ssr4 | Ssr4 | Signal sequence receptor, delta | 165 |
| Stx7 | Stx7 | Syntaxin 7 | 119 |
| Stxbp1 | Stxbp1 | Syntaxin binding protein 1 | 138 |
| Surf1 | Surf1 | Ab1-205 | 109 |
| Tacstd1 | Tacstd1 | Tumor-associated calcium signal transducer 1 | 105 |
| TCEB3 | Tceb3 | PREDICTED: elongin A isoform 1 | 130 |
| Tmed2 | Tmed2 | Transmembrane emp24 domain trafficking protein 2 | 204 |
| Tmed5 | Tmed5 | Transmembrane emp24 protein transport domain containing 5 | 185 |
| Tmed9 | Tmed9 | Transmembrane emp24 protein transport domain containing 9 | 106 |
| UBB | UBB | Ubiquitin B, isoform CRA_d | 106 |
| Vdac1 | Vdac1 | Voltage dependent anion channel | 267 |
| Ywhab | Ywhab | Tyrosine 3-monooxgenase/tryptophan 5-monooxgenase activation protein, beta polypeptide | 474 |
