# Supplementary material for: Comparative Proteomic Analysis of Lung Lamellar Bodies and Lysosome-Related Organelles
Source: PLoS One. 2011 Jan 26;6(1):e16482. doi: 10.1371/journal.pone.0016482 (PMC3027677; doi:10.1371/journal.pone.0016482)
Supplement: Table S4 — The LB proteome was analyzed for subcellular compartment enrichment by hypergeometric distribution using GO annotations of the human genome as reference background [48]. The Benjamini-Hochberg procedure was used to control family-wide false discovery rate ≤0.001 [49]. From the proteins identified in the current LB proteomics study, 16.7% (85 proteins) are known ER residents. The overlap of the LB and ER proteins is significant (P = 1.2×10^−14^). Gene Ontology Analysis was performed using public available web-based tool DAVID [48]. Overrepresented cellular components were selected at the threshold of False Discovery Rate (FDR) ≤0.005, minimum gene counts belonging to an annotation term ≥3% and Fold enrichment >2. Hypergeometric distribution was used to determine the degree of enrichment (PValue), FDR was determined using Benjamini-Hochberg procedure [49]. Fold enrichment measures the magnitude of enrichment, i.e. the % of proteins in LB list with a certain GO term versus the % of proteins with the same GO term in human genome (background). (DOC) [file pone.0016482.s006.doc]

| **GO Term** | **%** | **PValue** | **Fold Enrichment** | **Benjamini** |
| --- | --- | --- | --- | --- |
| GO:0042470~melanosome | 7.45 | 2.50E-30 | 11.81 | 1.20E-27 |
| GO:0048770~pigment granule | 7.45 | 2.50E-30 | 11.81 | 1.20E-27 |
| GO:0005788~endoplasmic reticulum lumen | 3.73 | 3.45E-10 | 6.57 | 8.70E-09 |
| GO:0044448~cell cortex part | 3.53 | 5.00E-09 | 6.00 | 9.21E-08 |
| GO:0044449~contractile fiber part | 4.31 | 5.17E-10 | 5.39 | 1.18E-08 |
| GO:0030017~sarcomere | 3.73 | 1.10E-08 | 5.36 | 1.81E-07 |
| GO:0043292~contractile fiber | 4.31 | 1.91E-09 | 5.03 | 3.99E-08 |
| GO:0030016~myofibril | 3.92 | 1.44E-08 | 4.98 | 2.23E-07 |
| GO:0015629~actin cytoskeleton | 9.22 | 4.68E-19 | 4.83 | 3.20E-17 |
| GO:0005938~cell cortex | 4.90 | 4.22E-10 | 4.74 | 1.01E-08 |
| GO:0005792~microsome | 6.67 | 2.29E-11 | 3.97 | 6.84E-10 |
| GO:0042598~vesicular fraction | 6.67 | 5.07E-11 | 3.86 | 1.43E-09 |
| GO:0016023~cytoplasmic membrane-bounded vesicle | 14.71 | 1.38E-23 | 3.77 | 1.66E-21 |
| GO:0031988~membrane-bounded vesicle | 15.10 | 4.54E-24 | 3.75 | 1.09E-21 |
| GO:0045177~apical part of cell | 4.71 | 1.19E-07 | 3.71 | 1.62E-06 |
| GO:0044445~cytosolic part | 3.92 | 2.28E-06 | 3.64 | 2.67E-05 |
| GO:0044432~endoplasmic reticulum part | 8.63 | 1.10E-12 | 3.51 | 3.77E-11 |
| GO:0031410~cytoplasmic vesicle | 15.88 | 2.65E-23 | 3.49 | 2.53E-21 |
| GO:0031982~vesicle | 16.47 | 5.19E-24 | 3.47 | 8.28E-22 |
| GO:0009898~internal side of plasma membrane | 7.65 | 6.02E-11 | 3.41 | 1.60E-09 |
| GO:0031252~cell leading edge | 3.33 | 3.70E-05 | 3.41 | 3.69E-04 |
| GO:0009986~cell surface | 8.24 | 1.95E-11 | 3.34 | 6.24E-10 |
| GO:0016324~apical plasma membrane | 3.14 | 8.86E-05 | 3.33 | 8.00E-04 |
| GO:0045121~membrane raft | 3.33 | 5.72E-05 | 3.29 | 5.37E-04 |
| GO:0009897~external side of plasma membrane | 3.92 | 1.20E-05 | 3.25 | 1.25E-04 |
| GO:0005912~adherens junction | 3.53 | 4.37E-05 | 3.21 | 4.18E-04 |
| GO:0048471~perinuclear region of cytoplasm | 6.27 | 5.17E-08 | 3.07 | 7.74E-07 |
| GO:0016323~basolateral plasma membrane | 4.31 | 1.40E-05 | 3.00 | 1.43E-04 |
| GO:0042175~nuclear envelope-endoplasmic reticulum network | 5.88 | 4.35E-07 | 2.92 | 5.63E-06 |
| GO:0070161~anchoring junction | 3.53 | 1.60E-04 | 2.90 | 0.001394359 |
| GO:0005789~endoplasmic reticulum membrane | 5.29 | 4.75E-06 | 2.78 | 5.17E-05 |
| GO:0005626~insoluble fraction | 16.27 | 3.05E-17 | 2.74 | 1.62E-15 |
| GO:0005624~membrane fraction | 15.69 | 2.45E-16 | 2.74 | 1.07E-14 |
| GO:0044463~cell projection part | 4.51 | 3.93E-05 | 2.72 | 3.84E-04 |
| GO:0000267~cell fraction | 20.00 | 8.59E-20 | 2.61 | 6.85E-18 |
| GO:0005840~ribosome | 3.92 | 2.93E-04 | 2.57 | 0.002374565 |
| GO:0000323~lytic vacuole | 3.73 | 6.39E-04 | 2.49 | 0.004849271 |
| GO:0005764~lysosome | 3.73 | 6.39E-04 | 2.49 | 0.004849271 |
| GO:0019898~extrinsic to membrane | 8.63 | 7.52E-08 | 2.46 | 1.06E-06 |
| GO:0005783~endoplasmic reticulum | 16.67 | 1.18E-14 | 2.45 | 4.70E-13 |
| GO:0005625~soluble fraction | 5.29 | 6.76E-05 | 2.39 | 6.23E-04 |
| GO:0005829~cytosol | 21.76 | 1.20E-17 | 2.31 | 7.16E-16 |
| GO:0042995~cell projection | 11.37 | 4.77E-09 | 2.30 | 9.13E-08 |
| GO:0005743~mitochondrial inner membrane | 4.90 | 3.04E-04 | 2.26 | 0.002426202 |
| GO:0005768~endosome | 4.90 | 4.65E-04 | 2.20 | 0.003589348 |
| GO:0012505~endomembrane system | 12.16 | 7.75E-09 | 2.19 | 1.33E-07 |
