# Supplementary material for: Comparative Proteomic Analysis of Lung Lamellar Bodies and Lysosome-Related Organelles
Source: PLoS One. 2011 Jan 26;6(1):e16482. doi: 10.1371/journal.pone.0016482 (PMC3027677; doi:10.1371/journal.pone.0016482)
Supplement: Table S5 — Comparison of LROs and LB proteome. (DOC) [file pone.0016482.s007.doc]

| Lysosome related organelle | Common Proteins | Percentage in Common |
| --- | --- | --- |
| Melanosome | 210 | 37.8 |
| Early stage of melanosomes (stage I & II) | 161 | 29.0 |
| Late stage of melanosomes (stage IV) | 110 | 19.8 |
| Exosome | 93 | 16.7 |
| Platelet | 86 | 15.5 |
| Endosome | 62 | 11.2 |
| Skin lamellar body | 55 | 9.9 |
| Lysosome | 51 | 9.2 |
| Synaptosome | 42 | 7.6 |
| Neuromelanin granule | 20 | 3.6 |
